# Sustainable Valorization of Biogenic Waste for Bone Repair and Regeneration: A Comprehensive Review of Eggshell and Aquatic Biomaterials

**DOI:** 10.3390/jfb17080417

**Published:** 2026-08-19

**Authors:** Shazah Waqar, Tamer A. E. Ahmed, Maxwell T. Hincke

**Affiliations:** 1 Department of Cellular and Molecular Medicine, Faculty of Medicine, University of Ottawa, Ottawa, ON K1H 8M5, Canada; swaqa005@uottawa.ca (S.W.); tahmed@uottawa.ca (T.A.E.A.); 2 Department of Innovation in Medical Education, Faculty of Medicine, University of Ottawa, Ottawa, ON K1H 8M5, Canada

**Keywords:** bone regeneration, eggshell, molluscan shell, critical-size defect, animal models, orthopedic implants

## Abstract

Bone loss represents a significant clinical burden that has driven the development of improved orthopedic graft substitutes. Although current grafting options, including autografts, allografts, and xenografts, have demonstrated considerable therapeutic potential, their widespread application is constrained by limitations such as donor scarcity, limited availability, and associated clinical risks. Consequently, biologically derived materials, including avian eggshells and marine bivalve shells, have emerged as promising alternative sources to produce bone precursor materials and next-generation bone graft substitutes. This review summarizes recent advances in avian eggshell- and marine shell-derived calcium carbonate (CaCO_3_) materials for bone regeneration and examines their preclinical evaluation in diverse animal models, including critical-size defects in calvarial, femoral, radial and mandibular bone. Relevant studies published over the past ten years were systematically analyzed, focusing on natural calcium carbonate systems derived from avian eggshell and marine shells, including oyster, mussel, clam, scallop, cockle, and sea urchins. A structured literature search was conducted using PubMed, Scopus, and Google Scholar to identify studies published between 2015 and 2025 investigating eggshell- and aquatic-derived biomaterials for bone repair and regeneration. Eligible studies were screened, and data were comparatively analyzed with respect to biomaterial source, scaffold fabrication, physicochemical characteristics, mechanical performance, biocompatibility, osteogenic potential, and the use of preclinical animal studies. Eggshell-derived biomaterials currently show the strongest translational evidence, while aquatic shell-derived biomaterials remain promising but underexplored for bone regeneration. Furthermore, this review critically examines the scientific, manufacturing, and regulatory challenges that must be addressed before clinical implementation.

## 1. Introduction

### 1.1. Overview of Bone Transplantation

Bone tissue is a vital structural component of all vertebrate species [1]. Damage to this mineralized connective tissue, whether through disease, injury, or natural defects, leads to difficulty sustaining normal functions, including movement, tissue growth, and mechanical strength, and is associated with pulmonary and joint complications [1].

**Figure 1:**
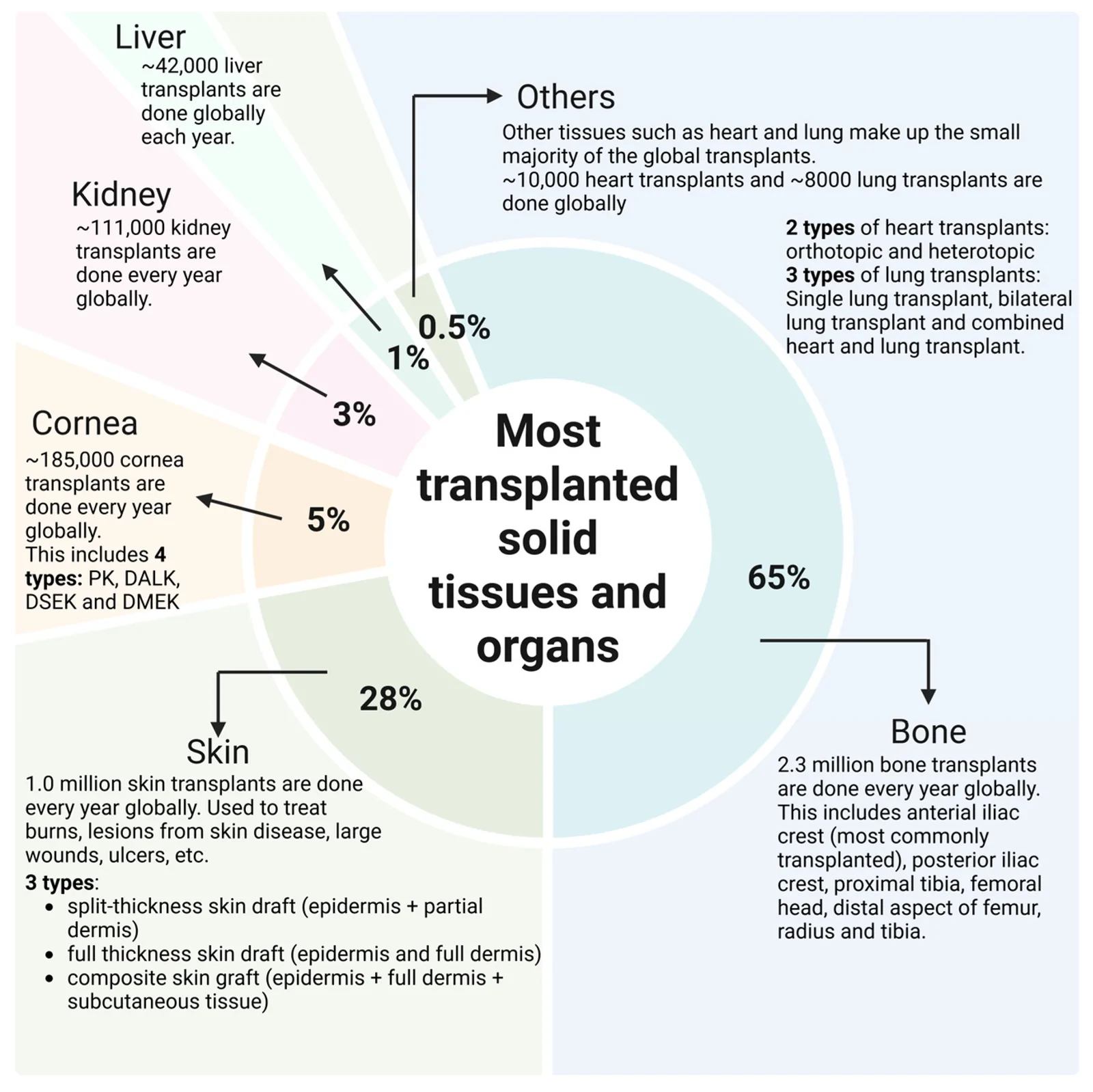
Schematic representation of the most transplanted solid tissues and organs globally per year with the highest part being dedicated to bone, followed by skin (3 types: split thickness skin draft, full thickness skin draft and composite skin graft), cornea (4 types: penetrating keratoplasty (PK), deep anterior lamellar keratoplasty (DALK), descemet endothelial keratoplasty (DSAEK) and descemet membrane endothelial keratoplasty (DMEK), kidney, liver and others such as heart and lungs. Original figure design and creation by S. Waqar.

The number of procedures performed to repair human-specific bone defects within the fields of orthopedics, dentistry and neurosurgery has been increasing by 13% per year globally, with 2.2 million bone graft procedures being completed each year, followed by skin, cornea, kidney, liver, heart and lung (Figure 1) [2,3,4,5,6,7,8,9]. This rising trend is partly attributable to the growing elderly population, a demographic particularly susceptible to multimorbidity and age-related skeletal conditions such as osteoporosis. The increasing incidence of traumatic bone injuries and musculoskeletal disorders has further exacerbated this burden [10]. The increased use of pre-implant grafting in craniofacial and spinal surgeries, and improved grafting material, has lowered the barrier for treating previously difficult orthopedic conditions, resulting in broader eligibility for surgical intervention and higher overall procedure rates [11,12]. In addition, greater survival rates after severe trauma and cancer resection have created a larger population requiring reconstructive procedures to restore segmental bone defects, while increased rates of revision arthroplasty have further increased clinical demand [11,13]. Modern imaging tools—including CT, MRI, and 3D surgical planning—now allow for earlier defect detection and more precise operations. These capabilities have directly driven an increase in total surgical procedures [14].

The global orthopedic implant market has an annual compound growth rate of 5.43%, is currently valued at 52.24 billion USD (2026) and is anticipated to reach 80.44 billion USD by 2034 [10]. An increasing demand for bone implants has driven researchers to identify and develop orthopedic implants composed of various materials.

### 1.2. Composite and Structure of Bone Tissue

Bone tissue is an essential component of the body’s structural framework and metabolic functions [15]. Beyond structural support, bone stores vital calcium and phosphate, houses blood cell production (hematopoiesis), and constantly remodels itself to adapt to mechanical and metabolic stress [16]. Bone tissue consists of cells, an organic matrix and an inorganic mineral phase. About 90% of the organic matrix of bone consists of collagen, with 10% proteoglycans, Gla-proteins (proteins containing gamma-carboxyglutamic acid), glycoproteins and phospholipids.

**Figure 2:**
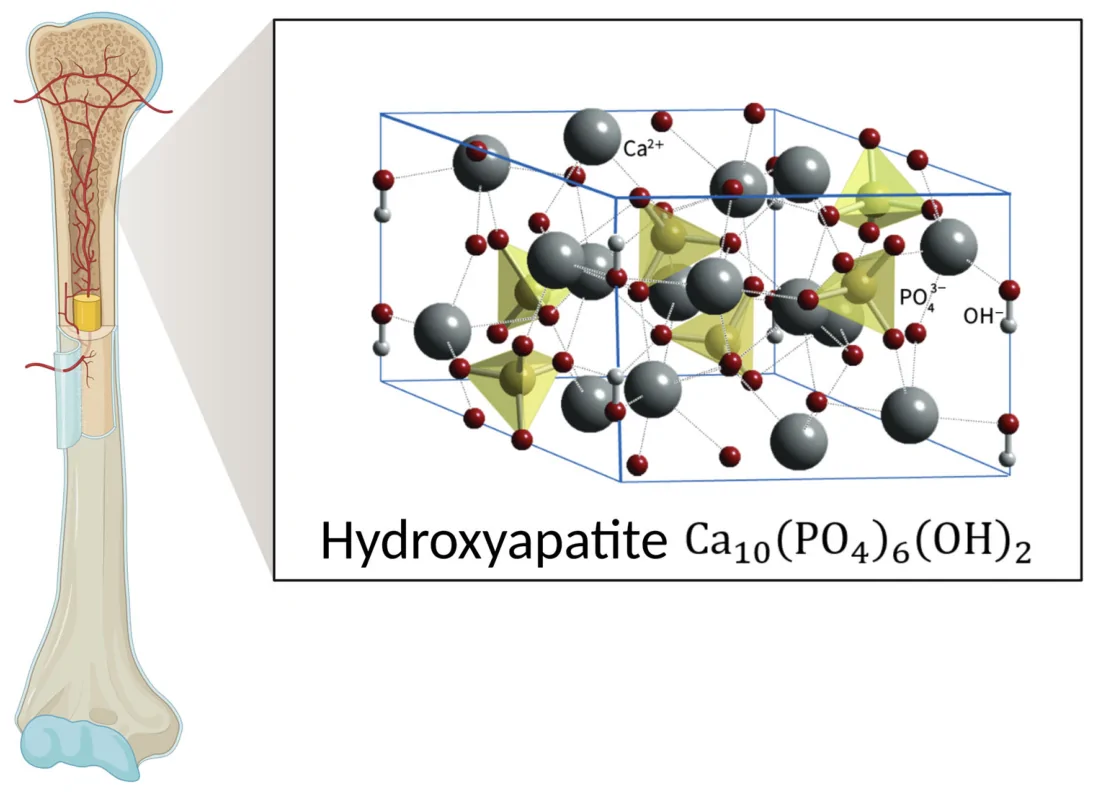
Schematic representation of mature long bone anatomy (**left**). The inset (**right**) depicts the crystal structure of hydroxyapatite (Ca_10_(PO_4_)_6_(OH)_2_) and the spatial arrangement of ions within the lattice. Original figure design and creation by S. Waqar.

Around 65–70% of bone is an inorganic mineral matrix composed of hydroxyapatite (HAp) crystals (Figure 2) [17]. Hydroxyapatite is a calcium phosphate (Ca_10_(PO_4_)_6_(OH)_2_) mineral that is primarily produced by osteoblasts, which secrete matrix vesicles in the extracellular matrix composed of Type 1 collagen and non-collagenous proteins [17]. These cells also produce Type 1 collagen, which serves as a substrate for mineral nucleation and crystal growth [17]. After matrix vesicles carrying phosphatases, calcium, and inorganic phosphate enter the collagen gap region, negatively charged non-collagenous proteins guide the growth of hydroxyapatite crystals beyond the fibril’s dimensions, which forms an interconnected, continuous cross-fibrillar pattern [18]. Within these vesicles, calcium and phosphate ions either remain amorphous or crystallize. Upon crystallization they burst through the vesicle membrane, forming nodules that become the nucleation sites for the hydroxyapatite crystals. These crystal particles continuously gather and align with the collagen fibrils to create mineralized lamellae that become the building blocks for bone tissue [18].

Bone tissue is primary composed of four types of cells: osteocytes, osteoblasts, bone lining cells and osteoclasts [15]. Osteocytes, osteoblasts and bone lining cells are derived from mesenchymal stem cells (MSCs) [15].

Osteoblasts are originally derived from MSC and later differentiate into osteocytes or bone lining cells or undergo apoptosis [19]. On the other hand, osteoclasts are derived from hematopoietic stem cells and differentiate into multinucleated bone-resorbing cells of the monocyte/macrophage lineage [19]. Osteoblasts make up 4–5% of the total bone cell population. They play a key part in bone formation, which is an integral part of skeletal development, growth and repair, as well as continuous remodeling throughout the organism’s life. Osteoblasts are derived from mesenchymal stem cells that differentiate through a carefully regulated network. This process relies on Wnt/β-catenin and bone morphogenetic protein signaling, and key transcription factors such as RUNX2 and osterix [19,20]. Mature osteoblasts are typically located along active bone-forming surfaces where they align in a cuboidal layer for the deposition of new extracellular matrix [19]. Their principal function is the synthesis and secretion of osteoid, a collagen-rich, unmineralized organic matrix composed of 90% type I collagen, along with non-collagenous proteins such as osteocalcin and osteopontin that regulate matrix organization and mineral nucleation [21]. Following matrix deposition and maturation, osteoblasts may undergo apoptosis, become quiescent bone lining cells, or differentiate into osteocytes once embedded within the mineralized bone [22].

Osteocytes are the most abundant bone cells (90–95%), which are derived from osteoblasts that have become embedded in the mineralized matrix through osteocytogenesis [23]. During this transition, they reduce matrix secretion, shrink in cell body size, and extend long dendritic processes [23]. Osteocytes are mature bone cells that live in small spaces within bone called lacunae and communicate with each other through a network of tiny channels [24]. They help keep bones healthy by controlling the balance between bone formation and bone breakdown, as well as regulating calcium and phosphate levels. Osteocytes can sense physical pressure placed on the bone, such as movement and weight-bearing activities [19]. In response, they release signaling molecules, including sclerostin, RANKL, and osteoprotegerin, which help control the activity of bone-forming cells (osteoblasts) and bone-resorbing cells (osteoclasts) [25]. Sclerostin slows down bone formation, but its production decreases when bones are physically loaded, allowing new bone to form. In contrast, aging or reduced physical activity increases sclerostin production and reduces bone formation [26].

Bone lining cells are thin, flat cells that develop from mature osteoblasts and cover about 80% of inactive bone surfaces [27]. They form a layer between the bone and surrounding tissues, where they help regulate interactions between bone and its environment. Functionally, they help maintain skeletal homeostasis by regulating bone remodeling and supporting the endosteal hematopoietic stem cell (HSC) niche [28]. Through cell–cell contact and secretion of regulatory factors, they maintain HSC quiescence and long-term regenerative capacity [29]. During remodeling, bone lining cells retract from the surface to permit osteoclast recruitment and activation. They also coordinate bone remodeling responses by forming a semipermeable barrier along the surface of the bone. This makes them a key player in ion exchange and the subsequent maintenance of mineral homeostasis [30]. This communication network extends to osteocytes, whose extensive connections are essential for sensing mechanical loading and directing bone remodeling processes [31].

Osteoclasts are primarily responsible for the resorption of bone tissue. This process is a key component of bone remodeling, including bone repair and release of calcium from bone stores to maintain mineral homeostasis. Bone resorption begins with the osteoclast creating an actin-rich sealing zone over the region of interest [32]. Within this sealing zone is a specialized ruffled border containing proton pumps, which lower the pH below 5, dissolving the bone mineral. Proteolytic enzymes, including cysteine proteinases and matrix metalloproteinases (MMPs), degrade the remaining collagen, while tartrate-resistant acid phosphatase (TRAcP) breaks down bone matrix proteins, allowing for complete removal of tissue [33,34]. Interactions at the mineral–collagen interface are critical for the initiation and organization of bone mineralization [35]. Type I collagen constitutes ~90% of the organic bone matrix (about 25–30% of total bone mass) and self-assembles into fibrils with a characteristic 67 nm D-periodicity, defined by alternating gap and overlap zones [36,37]. Osteoclast maturation and activation are regulated by macrophage colony-stimulating factor (M-CSF), RANKL, along with local inflammatory signals [20]. The periodic arrangement of collagen fibrils creates electrostatically distinct regions that serve as preferential sites for mineral deposition. These structural features guide the transformation of amorphous calcium phosphate into organized hydroxyapatite crystals, promoting crystal alignment parallel to the collagen fibrils and contributing to the mechanical properties of bone [35,36].

Non-collagenous proteins tightly control mineral expansion, based primarily on tissue-nonspecific alkaline phosphatase (TNAP), phosphocholine phosphatase 1 (PHOSPHO1), osteopontin (OPN), and osteocalcin (OCN) [37]. TNAP sits on the outer membrane of matrix vesicles and generates inorganic phosphate (Pi) by breaking down pyrophosphate (PPi). Because PPi naturally blocks mineralization, its destruction clears the path for crystal formation [38]. On the other hand, PHOSPHO1 operates inside the vesicle membrane, hydrolyzing phosphoethanolamine and phosphocholine to create the Pi needed for initial HAp nucleation [38,39]. A deficiency of either enzyme leads to hypomineralized bone, whereas loss of both causes embryonic lethality and a total failure of skeletal development [39].

Osteocalcin functions primarily as a regulator of crystal maturation. Through its calcium-binding gamma-carboxyglumtamic acid (Gla) residues, it interacts with the hydroxyapatite crystals and contributes to the regulation of crystal size, orientation and quality [36,40,41].

Osteopontin is a highly phosphorylated acidic protein that regulates mineral deposition and collagen organization in bone [37]. It binds calcium and attaches to hydroxyapatite crystals, where it inhibits further crystal growth. Osteoblasts release proteolytic enzymes, including MMPs and cathepsins, which degrade osteopontin and reduce its inhibitory effect, ensuring that mineralization occurs in specific bone regions [42].

### 1.3. Bone Healing and Repair Mechanisms

There are two primary mechanisms of bone healing: endochondral ossification and intramembranous ossification.

Endochondral ossification, responsible for most bone formation, is the process by which bone replaces hyaline cartilage during embryonic development, initiates long bone growth, and skeletal tissue repair [43,44]. It occurs within the hyaline cartilage of the epiphyseal growth plate at the ends of growing bones [43]. The process begins when mesodermal cells migrate to the site of bone development and form a condensed mesenchymal cell population [43]. These cells differentiate into chondrocytes, beginning at the center and progressing outward. Chondrocytes then proliferate and undergo hypertrophy, initially expressing collagen type X, followed by alkaline phosphatase (ALP) and other signaling molecules that produce a specialized extracellular matrix (ECM) serving as a template for calcification [44]. Hypertrophic chondrocytes release signaling factors along with matrix-remodeling mediators such as MMP-9 and RANKL, which promote vascular invasion from surrounding tissues and recruit osteoprogenitor cells, including osteoblasts and osteoclasts, that drive bone formation and remodeling [45]. This mechanism of bone repair is commonly studied in long-bone defect models, such as femoral and tibial defects, where healing occurs primarily through endochondral ossification.

Intramembranous ossification is the process where mesenchymal progenitor cells multiply and differentiate to directly form bone without first forming cartilage. [46]. This process occurs in flat bones such as the skull, face, jaw, shoulders, and pelvis [46]. It begins when mesenchymal cells cluster together in highly vascularized embryonic connective tissue [43]. These cells then become osteoblasts, which produce an osteoid matrix that later mineralizes to form woven bone. Osteoblasts trapped within this depositing matrix mature into osteocytes, establishing primary ossification centers made of calcified matrix and collagen fibers [47]. Blood vessels and hematopoietic tissue then invade the woven bone trabeculae to establish the bone marrow cavity. The primitive woven bone later remodels into structured lamellar bone, which is organized into a central cancellous core, and a dense outer cortical shell that provides mechanical load-bearing capacity [48]. Because craniofacial bones develop through this mechanism, calvarial and mandibular defect models are widely used in vivo to study bone regeneration and healing through intramembranous ossification [48].

For implantation to replace bone, host tissue (autograft) or orthopedic implants composed of metal, ceramic or a hydroxyapatite base are preferred. Regardless of the implant site (non-load bearing and load bearing), the implant design and material must be compatible with the host [49].

### 1.4. Bone Implantation

Various material sources are fundamental to orthopedic implant design, where factors such as implant size, type, and durability are critical determinants of performance [50]. It is important to differentiate between the osteogenic, osteoinductive, and osteoconductive properties of biomaterials. Osteogenic refers to the ability to promote bone-forming cell activity directly, osteoinductive describes the stimulation of progenitor cell differentiation toward the osteogenic lineage, and osteoconductive indicates the capacity to provide a supportive scaffold for bone growth [49,50].

**Figure 3:**
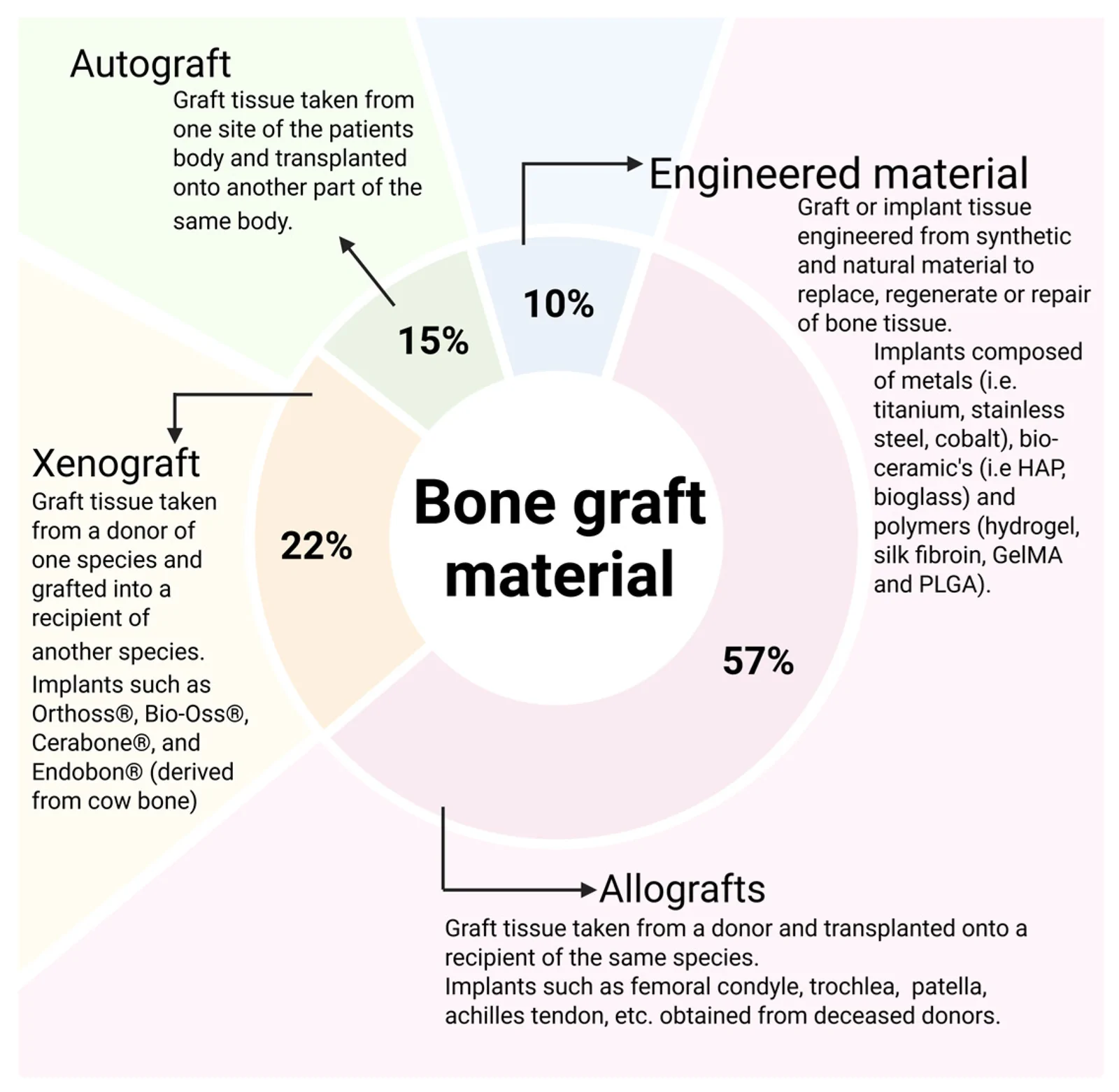
Schematic representation of an overview of implant materials (including biomaterials, biological tissue taken from a donor or patient) used for bone graft and their relative distribution in orthopedic applications. Original figure design and creation by S. Waqar.

Ideally, an autograft procedure can be performed to implant bone tissue taken from one part of the body into a deficient region. Bone samples are typically harvested from the iliac crest but can also be obtained from the posterior superior iliac spine, the distal femur, the proximal or distal tibia, the distal radius, or the olecranon (Figure 3) [49]. Autogenous bone grafts are beneficial because they are derived directly from the host, and are histocompatible, eliminating the risk of disease transmission and donor incompatibility (Figure 3) [51]. However, concerns such as donor site morbidity can significantly complicate extraction and recovery [52]. Autograft procedures have an 8.6% major complication rate, including prolonged wound drainage, sensory loss, infection, pain persisting for more than 6 months and in some cases require reoperation [53]. Minor complications, including superficial infection, minor wound problems, temporary sensory loss, and mild or resolving pain, were reported in 20.6% of patients [53]. If tissue from the host is unavailable, an autograft, which uses a donor’s tissue, is performed; however, this procedure poses a risk of transplant rejection, disease transmission, as well as difficulty finding a matching donor [51].

Depending on the implant region and the structural demands, a metallic alloy prosthesis –composed of titanium, copper and cobalt or various bioceramics—is widely used in both load-bearing and non–load-bearing applications to enhance mechanical stability, provide surface coatings of implant material, or function as a structural filler [54]. Despite their mechanical reliability, traditional metal and ceramic implants have distinct clinical challenges which can limit their long-term success [55].

### 1.5. Biomaterials for Bone Implantation

Biomaterials employed for bone tissue engineering encompass a diverse range of naturally derived, synthetic, and composite materials that are selected for their ability to mimic the characteristics of native bone (Figure 3) [49,50,54,55]. Naturally derived biomaterials, including protein- and polysaccharide-based polymers, are widely valued for their biocompatibility and ability to support cell adhesion and tissue regeneration. Protein-based polymers such as silk fibroin possess intrinsic cell-binding motifs that promote cellular attachment and proliferation [56]. Similarly, carbohydrate-based polymers including chitosan and alginate have been applied in various applications due to their low immunogenicity, tunable properties, and ability to form porous scaffolds that facilitate diffusion, vascularization, and new tissue formation [57,58,59,60].

In contrast, synthetic biomaterials such as polycaprolactone (PCL), polylactic acid (PLA), polyglycolic acid (PGA), and poly(lactic-co-glycolic acid) (PLGA) provide superior mechanical strength and reproducible manufacturing, making them attractive for load-bearing applications [61]. However, these materials generally lack intrinsic bioactivity and cell recognition sites, often requiring surface modification or incorporation of bioactive components to enhance cellular responses [61].

To overcome the limitations associated with individual material classes, composite biomaterials have emerged as a promising strategy by combining the biological advantages of natural polymers with the mechanical stability and osteoconductivity of inorganic mineral phases. Common examples include polymer–ceramic composites incorporating hydroxyapatite (HAp), β-tricalcium phosphate (β-TCP), bioactive glass, or calcium carbonate-based materials, which more closely resemble the composition and structure of bone while improving mechanical performance and osteogenic potential [56,60,62,63]. Despite the excellent biological properties of natural polymers, their limited mechanical strength and osteoconductive capacity often necessitate reinforcement with mineral components, particularly for load-bearing orthopedic applications.

Calcium-rich biomaterials derived from natural sources, including demineralized bovine bone, avian eggshells and molluscan shells, represent alternatives due to their compositional similarity to the inorganic phase of bone [64]. These materials have been investigated as bone graft substitutes either in their native form or following conversion into calcium phosphate-based ceramics such as hydroxyapatite or calcium phosphate, or as reinforcement within polymeric scaffolds [65,66]. Incorporation of these calcium-based biomaterials improves the mechanical stability of scaffolds, increases osteoconductivity and promotes mineral deposition during bone regeneration [67]. Furthermore, their widespread availability and derivation from poultry or marine waste streams support the development of cost-effective and sustainable biomaterials [68]. In 2024 alone, an average of 10 million metric tons of ES waste was discarded into landfills worldwide [58]. In addition, over 10 million tons of molluscan shells are produced each year, of which over 70% is represented by oyster, clam, scallop and mussel shells. These shells account for most of the organism’s weight and are largely discarded [59]. By combining the biological functionality of natural polymers with the structural and osteogenic advantages of calcium-rich materials, composite scaffolds can more closely replicate native bone, thereby improving host tissue integration and supporting new tissue formation. Derived mostly from sustainable, calcium-rich sources, their excellent biocompatibility and osteoconductive traits make them highly effective for bone regeneration [62,69].

Nevertheless, despite their considerable promise, several challenges continue to limit the clinical translation of shell-derived biomaterials. Variations in composition arising from species, geographical origin, diet, and processing methods can influence their physicochemical properties and biological performance, highlighting the need for standardized collection, purification, and fabrication protocols [56]. In addition, scalable manufacturing processes that ensure consistent quality, reproducibility, sterilization, and regulatory compliance remain underdeveloped [56,59,62,69]. Addressing these challenges will be essential to fully realize the potential of waste-derived calcium-rich biomaterials as reliable and commercially viable alternatives to conventional synthetic and mined biomaterials.

Despite growing interest in calcium carbonate-based biomaterials for skeletal repair, existing reviews have largely focused on individual biomaterial classes, synthesis techniques, or specific natural sources. A comprehensive comparison of avian- and marine shell-derived biomaterials and their performance in preclinical models of bone regeneration remains lacking. This review addresses this gap by critically evaluating studies published over the past decade that investigate naturally derived shell-based biomaterials for orthopedic applications. Emphasis is placed on biomaterial processing, physicochemical properties, osteogenic performance, and regenerative outcomes across preclinical animal models, while also examining the translational potential and current challenges that must be overcome before clinical implementation. The review is structured to first summarize the biological principles of bone repair, followed by the preparation and characterization of shell-derived biomaterials, then a comparative assessment of avian and marine shell-based constructs in bone regeneration, and finally a discussion of emerging opportunities and future directions for clinical translation.

## 2. Bioceramics Derivatives from Avian Eggshell

The following section includes synthesis, characterization, and preliminary in vitro biological evaluation of bioceramics derived from eggshell.

### 2.1. Composition and Structure of Avian Eggshell

Avian eggshell (ES) is a calcium-rich, naturally derived bioceramic whose precise structural details vary with species (i.e., ES thickness), although physicochemical properties are similar. While all avian ESs are composed of calcium carbonate (CaCO_3_), in the form of calcite, the percentage of trace ions, organic material and thickness varies depending on the avian species. The most-studied avian source is the chicken ES due to its consistent composition and widespread availability as a major by-product of the food industry [55]. Moreover, its abundance as an agricultural waste product, low cost, and suitability as a biocompatible calcium source make it a well-studied biomaterial in bioceramic research [55].

Chicken ES is predominantly composed of inorganic material, as mineralized calcium carbonate in the calcite polymorph (96%), including trace levels of magnesium (Mg^2+^), phosphate (PO_4_^3−^), and various minor elements, while the remaining fraction is an organic matrix rich in proteins [55,57]. The chicken ES is thin (~0.35 mm) and morphologically comprises of three distinct layers [57]. The innermost layer is called the mammillary layer and is composed of cone-shaped points that anchor the shell to the underlying eggshell membrane. This is also the first site of a mineral deposit during shell development [57]. Above this is the palisade layer, the thickest portion of the shell, composed of tightly packed, columnar crystals that provide the majority of the shell’s strength [57]. A transitional layer (vertical crystal layer, VCL) between the cuticle and palisade layer increases shell strength [57]. Finally, the outer proteinaceous cuticle layer on the shell surface is a protective barrier against microbial invasion and environmental damage and plays a role in regulating gas and water exchange by occluding the respiratory pores that traverse the eggshell. Each of these ES layers exhibits a finely tuned combination of mineral and organic components, which together ensure the shell is both mechanically strong and physiologically functional [64].

### 2.2. Eggshell Collection and Sanitization

Eggshells (ESs) must be thoroughly cleaned and sterilized before use as biomaterial precursors. This includes the removal of eggshell membrane (ESM), pathogens, organic residues, and other surface contaminants that can compromise biomaterial purity and performance [65]. Initial cleaning typically involves boiling or rinsing with deionized (DI) water, phosphate-buffered saline (PBS), or detergent to remove surface debris [66,67,68]. Additional sterilization may include ethanol treatment combined with ultrasonication to eliminate residual organic matter [59]. Complete ESM removal is commonly achieved using chemical treatments such as bleaching solutions, 1 M acetic acid, 1 M nitric acid, or calcium hydroxide, while thermal processing (>450 °C), plasma etching, and enzymatic digestion with protease have also proven effective in producing a clean mineral substrate suitable for further processing [56,59,62,69].

### 2.3. Shell-Derived Calcium Carbonate Conversion Strategies

**Figure 4:**
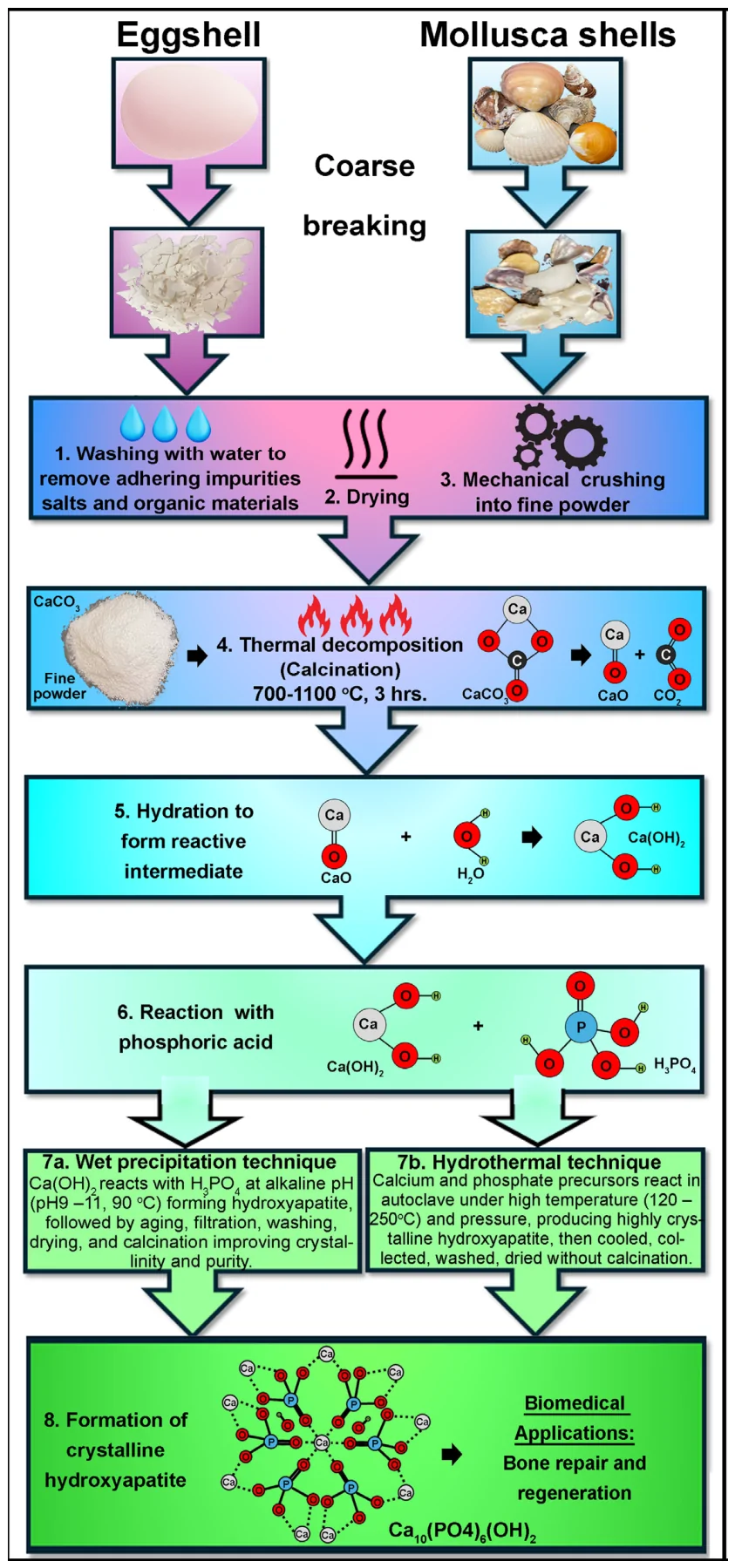
The sequential transformation of eggshell and bivalve shells into hydroxyapatite is described in stages for wet precipitation (7a) and hydrothermal synthetic (7b) approaches. Original figure design and creation by T. Ahmed.

Calcium carbonate-based bioceramics, such as those derived from avian and marine shells, have gained increasing attention for their potential osteoconductive nature due to their capacity to be converted into bioactive calcium phosphate phases suitable for the promotion of bone regeneration [55]. Both avian eggshells and bivalve shells have been demonstrated to be a sustainable precursor for the synthesis of HAp [62,69,70,71,72]. The technique for converting this material into HAp is highlighted in Figure 4.

The process begins with thoroughly cleaned shells, which are mechanically crushed into a fine powder to facilitate subsequent processing and improve reaction efficiency. If the final implant is intended to retain its native calcium carbonate (CaCO_3_) composition, the material is used directly following particle size optimization and any required sterilization procedures [66]. When hydroxyapatite (HAp) is required, the shell powder undergoes calcination through thermal decomposition, typically at temperatures ranging from 700 to 1100 °C (Figure 4, step 4) [68]. This process converts calcium carbonate into calcium oxide (CaO) while releasing carbon dioxide (CO_2_) as a by-product. The resulting calcium oxide is subsequently hydrated to produce calcium hydroxide (Ca(OH)_2_), which serves as the calcium precursor for hydroxyapatite synthesis (Figure 4, step 5) [58]. Parameters such as the Ca/P molar ratio, pH, reaction temperature, mixing rate, and ageing time are carefully regulated to promote the formation of stoichiometric hydroxyapatite while minimizing the generation of secondary calcium phosphate phases [58,59]. In the final stage, calcium hydroxide is reacted with a phosphate source, most commonly phosphoric acid (H_3_PO_4_), to form hydroxyapatite [62]. This conversion is primarily achieved using either wet precipitation, which is the most widely reported method in the literature due to its simplicity and cost-effectiveness, or hydrothermal synthesis, which is often employed to produce hydroxyapatite with enhanced crystallinity, phase purity, and more controlled particle morphology (Figure 4, steps 7a and 7b) [56,62]. Overall, these processing methods enable the conversion of biogenic shell waste into calcium carbonate- and hydroxyapatite-based biomaterials with properties that can be tailored for bone tissue engineering and orthopedic applications.

### 2.4. Eggshell-Derived Biomaterials for Bone Regeneration

Neunzehn and coworkers [66] studied the effects of adding crushed ES powder (50 µm) to in vitro cell cultures of bovine osteoblasts. They found that cells supplemented with ES showed the highest increase in proliferation, with osteoconduction advancing further than the controls (particularly noted by the denser extracellular matrix). In addition to this, these cells demonstrated elevated levels of osteonectin and osteocalcin, two non-collagenous bone matrix proteins involved in mineralization, as well as collagen I, the primary structural protein of the bone extracellular matrix. Metabolic assays showed that cells treated with eggshell (ES) supplementation had higher activity and oxygen consumption compared to untreated controls and pure calcium carbonate (CaCO_3_), indicating a stronger osteoconductive response. These results suggest that ES powder can enhance osteoblast function and may be useful for bone graft applications [66].

Huang and coworkers [67] used ball-milled calcium carbonate (crushed ES in powder form) to reinforce and enhance the performance of a magnesium-yttrium (Mg-Y 3.5 wt%) composite alloy. ES was incorporated at 1, 3, and 5 wt% using resistance casting, resulting in homogeneous reinforcement distribution. The alloy reinforced with 3 wt% ES powder demonstrated the most favorable mechanical compatibility and similarity to cortical bone, with a compressive strength of 161.19 MPa and elongation of 17.53%, overall reducing the risk of stress shielding for load-bearing bone implant applications [67].

Beyond using ES-derived calcium as a precursor, researchers have utilized methods to convert calcium carbonate into hydroxyapatite. Amiruddin & Noor [68] converted ES into hydroxyapatite (HAp) using a wet precipitation method followed by two-stage calcination (900 °C and 1000 °C) and evaluated the effects of pH and calcination temperature on material properties. Fourier-transform infrared (FTIR) spectroscopy showed the characteristic phosphate and hydroxyl peaks of hydroxyapatite. Scanning electron microscopy (SEM) indicated that both pH and calcination temperature significantly affected particle shape, with higher temperatures leading to greater crystallinity. Overall, these results suggest that ES-derived hydroxyapatite has good phase purity and suitable microstructural properties for use in bone implant coatings [68].

ES-derived calcium materials have also been integrated into bioactive composite systems for coatings, often in combination with polymers or dopants to improve osteoconductive and mechanical properties further. Sekar and Lee [58] demonstrated that nanohydroxyapatite (nHAp) synthesized from ES, combined with chitosan, can be engineered into orthopedic implant coatings. ES-derived nHAp was doped with europium and yttrium (substituted for calcium) and exhibited high phase purity, crystallinity, and strong bonding with the biopolymer matrix, resulting in coatings with improved mechanical stability and surface bioactivity. These composites showed high cell viability (~95%) in fibroblast and osteoblast-like cells grown on coated stainless-steel implants. Increased apatite formation was also observed in simulated body fluid (SBF), confirming good in vitro biocompatibility [58].

Santosh Kumar et al. [59] produced nano-hydroxyapatite from eggshells and used it to make porous bone scaffolds using a foam replica method. Tests such as SEM, XRD, and FTIR confirmed that the material formed crystalline nanosized particles (about 75–83 nm), contained small amounts of bone-related ions, and had a well-connected porous structure (100–550 μm). Mechanical testing showed a compressive strength of 3–5.5 MPa, similar to trabecular bone. Laboratory studies also showed controlled degradation, good water affinity, antibacterial properties, and strong compatibility with cells. Cell viability of MG63 and L929 cells was above 75% after 24 h and increased to over 90% over time. Overall, these results indicate that ES-derived nHAp scaffolds have suitable structural, mechanical, and biological characteristics for application as sustainable synthetic bone substitute materials [59].

Similarly, Palakurthy and colleagues [69] fabricated an ES-derived bioceramic for bone regeneration by calcining ES at 900 °C and combining it with silica via a modified sol–gel route to produce β-wollastonite with varying strontium content. β-wollastonite, a crystalline calcium silicate (CaSiO_3_), showed strong bioactivity and the ability to bond with bone in laboratory tests. When placed in simulated body fluid, ES-derived ceramics quickly formed a layer of hydroxyapatite. pH monitoring showed that the material degraded in a controlled way that is compatible with physiological conditions. MTT tests using MG-63 osteoblast-like cells confirmed that the material is biocompatible. Strontium-substituted versions further improved cell growth and showed no toxic effects [69].

Gutiérrez-Prieto et al. [62] showed that a silicon-doped hydroxyapatite scaffold made from eggshells and reinforced with PLGA supported strong osteoblast attachment and viability in vitro. Osteoblasts grown on the HAp/Si/PLGA composite had high survival rates (~96%) and formed a continuous cell layer, indicating good interaction with the material. The scaffold also had a porous, interconnected structure that allowed cells to grow and move through it easily. PLGA improved the mechanical stability of the scaffold, overcoming the brittleness of silicon-doped hydroxyapatite. Overall, this composite performed better in supporting osteoblast growth than eggshell-derived HAp/PLGA and bovine bone/PLGA controls [62].

Beyond reinforcing load-bearing metallic implants, ES-derived calcium materials have also been incorporated into injectable, polymer-based scaffolds designed for bone regeneration. Chuysinuan et al. [56] developed an injectable silk fibroin–alginate hydrogel incorporating hydroxyapatite synthesized from ES for bone tissue engineering. ES-derived HAp was obtained through calcining crushed shells at 900 °C, followed by chemical precipitation. Physiochemical analysis demonstrated scaffolds with ES-derived HAp to have reduced pore size and porosity, a sufficient degradation rate, as well as fulfilling the requirements of compressive strength needed for non-load-bearing defects. In vitro evaluation using MC3T3 E2 pre-osteoblasts showed hydrogels to be supportive of cell proliferation, adhesion and differentiation, as well as non-cytotoxic. Increased activity of bone marker enzymes such as alkaline phosphatase reflected osteoconductive potential [56].

Calvert et al. [60] studied the use of hydrogel (alginate-chitosan) based scaffolds that incorporate ES and nanotextured eggshell particles (NTES—treated with phosphoric acid) for bone regeneration. Compared to particle-free scaffolds, ES- and NTES-containing scaffolds showed higher porosity, better stability in liquid and enhanced resistance to degradation. In vitro testing using seeded human mesenchymal stem cells demonstrated greater cell retention and viability up to 21 days. Elevated expression of bone-related marker enzyme alkaline phosphatase indicated the onset of early cell differentiation [60].

Ma et al. further demonstrated that amorphous calcium phosphate (ACP) derived from eggshell exhibits osteoconductive properties using the THP-1 human monocytic cell line [63]. The ES-derived ACP exhibited improved physicochemical stability, greater resistance to recrystallization, and higher cytocompatibility relative to control ACP. It also increased cell differentiation, as shown by increased expression of such as osteopontin and osteocalcin, enhanced mineralized nodule formation, and elevated extracellular matrix production, especially collagen type I [63].

### 2.5. Current Status of ES-Derived Biomaterials for Bone Regeneration

The studies reviewed demonstrate several consistent trends in the use of ES-derived biomaterials for bone regeneration. First, ES-derived calcium sources repeatedly enhanced osteoblast proliferation, viability, and expression of bone markers, indicating an osteoconductive effect across both calcium carbonate-based and hydroxyapatite-based systems [56,58,59,60,65,66,67,68,69]. Second, converting ES into hydroxyapatite or other bioactive calcium phosphate phases generally produced superior biological performance compared with raw eggshell powder alone, particularly in terms of apatite formation, cell attachment, and bone regeneration [59,67,68]. Materials enriched with biologically relevant ions, including magnesium, often demonstrated further improvements in osteogenesis and scaffold bioactivity [67]. Eggshell-derived calcium was used as the precursor for synthesizing strontium-substituted β-wollastonite systems [69] and silicon-doped hydroxyapatite/PLGA composites [62], both of which demonstrated enhanced in vitro osteoconductivity and excellent cytocompatibility. These findings suggest that the most promising eggshell-derived biomaterials are not necessarily unmodified hydroxyapatite but rather composites that combine osteoconductivity with controlled degradation and enhanced biological signaling. Despite these encouraging results, several limitations remain. These studies are conducted in vitro, with relatively short follow-up periods and experimental designs that make direct comparison difficult. Therefore, while eggshell-derived biomaterials consistently demonstrate osteoconductive potential and represent a sustainable alternative to conventional calcium phosphate sources, further standardized in vivo studies and controlled clinical investigations are required to determine which formulations provide the optimal balance of bioactivity, mechanical stability, degradation behavior, and translational feasibility.

## 3. Bioceramics Derivatives from Molluscan Shell

The following section includes synthesis, characterization, and preliminary in vitro biological evaluation of bioceramics derived from aquatic shells.

### 3.1. Composition and Structure of Bivalve Mollusca Shells

**Figure 5:**
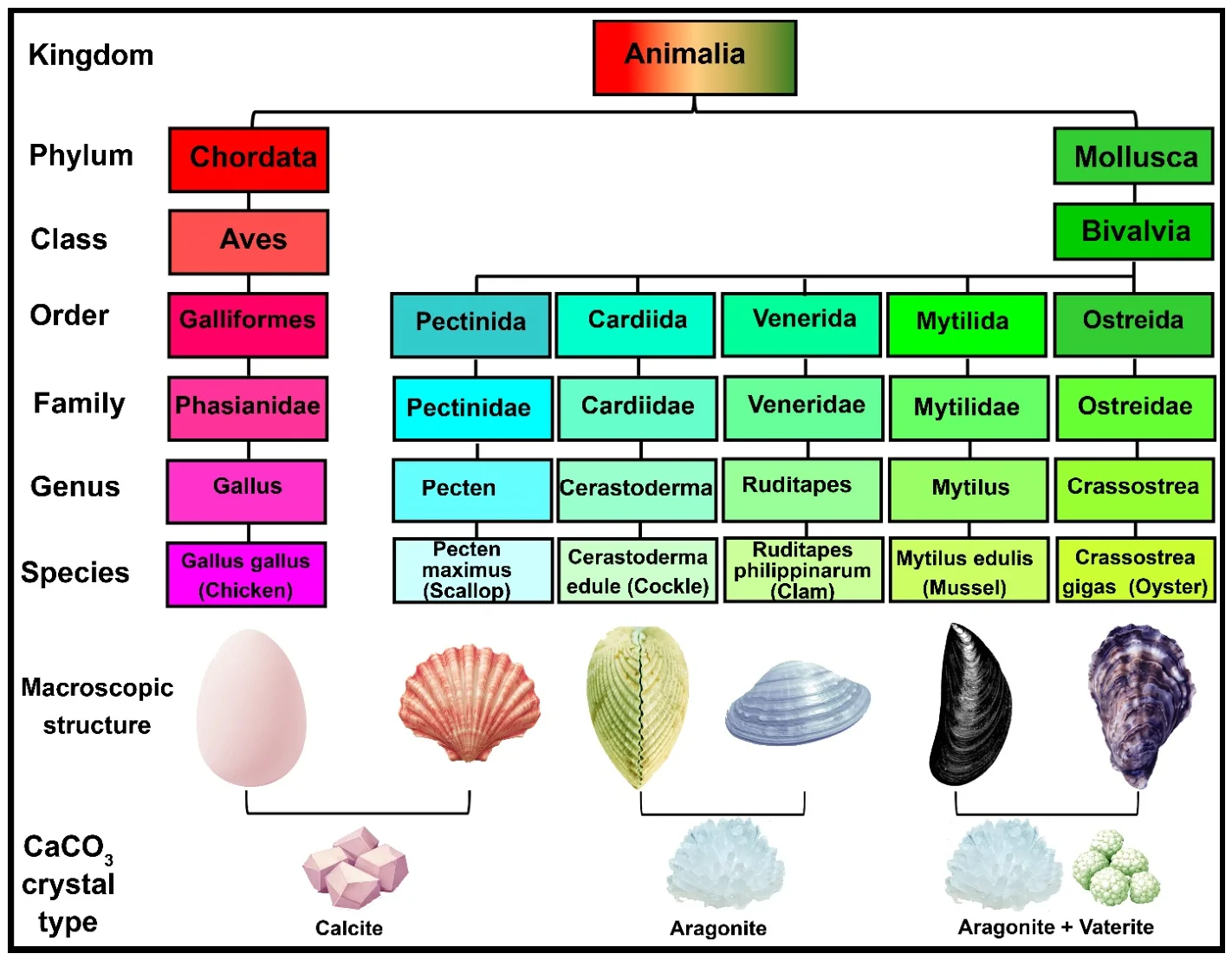
The taxonomic of selected bivalve molluscan shells and avian eggshells relevant to biomineral-based applications for orthopedic purposes. The classification ranges from kingdom to species, with depiction of the relevant calcium carbonate polymorphs (calcite, aragonite, vaterite). Original figure design and creation by T. Ahmed.

Bivalves are a class of mollusks (Class Bivalvia) characterized by a soft-flesh body enclosed within two hinged shells, more commonly known as valves (Figure 5) [73]. They are mostly aquatic, living in marine and freshwater environments, and include, but are not limited to, well-known invertebrates such as oysters, mussels, scallops, clams, and cockles (Figure 5) [73]. Bivalve shells are primarily (95–99%) composed of calcium carbonate (CaCO_3_), which occurs in the crystalline forms of aragonite and calcite, depending on the species and shell layer (Figure 5) [73,74]. Bivalve shells also contain a minor organic fraction containing proteins and polysaccharides (approximately 1–5%), which acts as an organizing matrix for mineral growth and contributes to overall toughness and flexibility [75]. Most shells are made of three main layers: the outer periostracum, a thin organic layer that protects against chemical and physical damage; the prismatic layer, made of tightly packed calcite or aragonite crystals that provide strength; and the inner nacreous layer (mother-of-pearl), made of aragonite platelets arranged in a brick-and-mortar structure that increases shell toughness [76,77]. In most bivalves, the periostracum, the outermost organic shell layer, is secreted by basal cells located at the base of the periostracal groove along the inner surface of the outer mantle fold. [78,79]. It is the thinnest layer, composed of protein-based materials rich in amino acids such as cysteine and methionine, and is responsible for protecting the shell from environmental stressors, including acidic surroundings, shell dissolution, and mechanical stress [79,80]. Removal of the periostracum during preprocessing is critical, as its organic composition influences thermal decomposition and sample purity [80]. Beneath the periostracum lies a mineralized prismatic layer made up of closely packed aragonite or calcite crystals that are oriented perpendicular to the shell surface [80,81]. These crystals are partitioned by a thin organic membrane, which controls crystal development and helps preserve overall structural integrity [81]. The vertical direction of crystal growth makes this layer moderately resistance to fractures and slightly flexible. Attached firmly to the prismatic layer is the nacreous layer, the bottom-most layer that is in contact with soft flesh [82]. This layer consists of small, flat aragonite crystals densely packed into a diagonal sheet-like structure, making it extremely rigid and least prone to fractures [82]. The sizes of these layers vary depending on the bivalve species and the geographical environments that they are found in [82].

Bivalves such as oysters, clams, and mussels have shells comprising all three layers, whereas others like scallop shells and cockle shells contain only the outer prismatic layer and the inner nacreous layer [79,80,81,82]. This combination of mineral rigidity and organic reinforcement gives bivalve shells their remarkable mechanical properties, making them highly suitable as natural precursors for biomaterials [82].

### 3.2. Bivalve Shell Collection and Cleaning

Bivalves for research and biomaterial development can be obtained from a variety of sources. In laboratory-scale studies, bivalve shells are commonly collected from various geographical locations, including coastal waters, mangroves, and river mouths [83]. Bivalve shells must undergo thorough pre-processing prior to their use as implants or as precursors for biomaterial synthesis, in order to eliminate residual organic matter, adhered tissue, sediments, and biofouling organisms such as algae [83].

A variety of cleaning and decontamination methods have been described for the elimination of smaller particles and surface-adhering impurities, which range from mechanical approaches such as hand scrubbing using water [84] and brushes [83], to more sophisticated techniques like ultrasonic cleaning [85,86]. Thermal and chemical strategies are frequently used, including boiling water treatments for sterilization and the application of mild chemical detergents to remove residual organic matter [70]. After cleaning, shells are dried or calcined at high temperatures (usually above 300 °C) to remove remaining organic material and eliminate microbial contamination [87]. Proper cleaning is important because impurities can interfere with later processing steps and reduce the chemical purity and biological performance of the final biomaterial.

### 3.3. Clam Shell-Based Biomaterials

Murugiah et al. [88] studied hydroxyapatite (HAp) made from hard clam and blood cockle shells. The powders were analyzed before and after sintering at 900 °C using XRF, XRD, FTIR, and SEM. Both shell types were mainly composed of calcium carbonate (>97 wt%). After heating, they formed crystalline hydroxyapatite with the expected phosphate, carbonate, and hydroxyl groups. Heat treatment increased crystallinity and caused particles to fuse. Although the Ca/P ratios were slightly higher than ideal HAp, both materials closely resembled pure hydroxyapatite [88].

Hassanein et al. [89] explored the synthesis, structural properties, and bioactivity of HAp obtained from clam shells for potential bone-related applications. The resulting HAp powder was analysed using XRD, FTIR, SEM, and energy-dispersive X-ray spectroscopy (EDS) to assess phase composition, crystallinity, surface morphology, and elemental makeup. The successful formation of crystalline hydroxyapatite demonstrated the expected phosphate and hydroxyl groups, with Ca/P ratios approaching that of stoichiometric HAp. SEM imaging showed agglomerated particles ranging from the micro- to nanoscale dimensions. In vitro testing in simulated body fluid (SBF) revealed the development of an apatite layer with strong potential for osteoconductivity [89].

Building on the development of clam shell-derived HAp powders, several studies [90,91] have explored how these powders can be processed into coatings and dense bioceramics suitable for orthopedic applications. In one such study, Hussain and co-researchers [90] investigated the influence of heat treatment on the clam shell-derived HAp on Ti-6Al-4V alloy substrates. HAp was produced using CAO obtained from thermally treated clam shell powder, which was subsequently reacted with calcium phosphate under hydrothermal conditions at 700 °C to 1100 °C to obtain HAp powder. The powder was used in spray-drying processes for a potential orthopedic coating. The coating was heat-treated after spraying at temperatures between 500 °C and 900 °C to study how temperature affects phase formation and stability, with 800 °C identified as the best condition. Microstructural and surface analysis showed that higher temperatures increased the crystallinity of hydroxyapatite (HAp). Mechanical tests also showed improved hardness and wear resistance. Overall, hydroxyapatite from clam shells can be used for strong implant coatings, but careful control of heat treatment is needed to balance stability and mechanical performance for orthopedic and dental applications [90].

Ng et al. [91] studied hydroxyapatite (HAp) bioceramics made from clam shell waste to evaluate their use in biomedical applications. The material was compacted and sintered at 1200–1300 °C to test phase stability and mechanical properties. The best results were obtained at 1250 °C. Overall, the clam shell–derived HAp showed good structural strength and suitable mechanical properties, indicating potential for use in bone-related biomedical applications [91].

In addition, clam shell-derived hydroxyapatite has been incorporated into polymer-based composite systems to improve mechanical strength and wear resistance for bone substitute materials. Oladele et al. [92] reported that incorporating this HAp into an epoxy matrix significantly enhanced composite performance. At filler loadings of approximately 12–15 wt.%, the composites showed optimal improvements in flexural strength (up to ~61 MPa), hardness (~61 HRA), impact resistance (~16 J), and wear resistance compared with neat epoxy. The study identified clam shell-derived HAp as a sustainable and cost-efficient reinforcing ceramic, with strong potential for load-bearing orthopedic and dental applications where mechanical durability and wear resistance are essential [92].

Saravanan and coworkers [61] developed nano-hydroxyapatite (nHAp) composites from clam shell, poly(lactic-co-glycolic acid) (PLGA) and chitosan for potential bone healing applications. The nano-hydroxyapatite (nHAp) was produced using a wet chemical precipitation method. Calcium oxide from clam shells was dissolved in nitric acid and then reacted with diammonium hydrogen phosphate at a Ca/P ratio of 1.67. The resulting precipitate was calcined at 900 °C for 3 h to form crystalline nHAp. Composite materials made with PLGA and chitosan were analyzed using XRD, FTIR, SEM, EDX, CHNS, and crystallinity tests. These analyses confirmed successful nHAp formation and showed that adding polymers increased carbonate content and reduced crystallinity, making the material more similar to natural bone mineral. Among the composites, nHAp/Chitosan exhibited the smallest particle size, the highest carbonate content, and the most favorable mechanical behavior, along with strong antibacterial activity against Staphylococcus aureus and Escherichia coli. Hemolysis testing further demonstrated that nHAp/Chitosan was non-hemolytic and hemocompatible, while in vitro apatite formation after 14 days indicated promising bioactivity [61].

Similarly, Sindhya et al. [84] demonstrated that nHAp synthesized from clam shells can produce a biocompatible biomaterial suitable for bone and dental applications. The clam shell powder was calcined, followed by nitric acid dissolution and co-precipitation with diammonium hydrogen phosphate to yield nHAp at a Ca/P ratio of ~1.68. The polymer capping agents PEG and PVA were added during synthesis to control crystal growth as well as improve surface characteristics. Of the different formulations, PVA-capped nHAp exhibited the smallest crystallite size (~32 nm), with improved thermal stability and uniform morphology. In vitro evaluations showed rapid apatite layer formation in simulated body fluid, strong antibacterial activity, and excellent hemocompatibility (hemolysis ~1.07%), indicating high biological safety and biocompatibility for potential use in in vivo applications [84].

Alterations such as doping and polymer capping have been studied for the purposes of optimizing the performance of clam shell-derived HAp. Pal and coworkers [70] investigated the effectiveness of strontium-doped hydroxyapatite (Sr-HAp) derived from clam shell as a suitable alternative for bone-related biomedical applications. Hydroxyapatite was synthesized using a hydrothermal method, followed by reaction with diammonium hydrogen phosphate and substitution with strontium, and then sintered at 1200 °C. Mechanical testing showed that increasing strontium content improved density, hardness, fracture toughness, and elastic modulus. In vitro studies using MC3T3-E1 mouse osteoblasts showed that all samples were non-cytotoxic. The highest cell proliferation occurred at moderate strontium levels (≤50%), while higher levels reduced proliferation. Overall, strontium-doped hydroxyapatite from clam shells improved mechanical properties while maintaining good cell compatibility, suggesting potential for orthopedic applications [70].

The porous structure and degradation characteristics of clam shell–derived materials have also been studied to assess their suitability for promoting bone ingrowth and tissue remodeling. Wu et al. [71] reported that porous calcium phosphate granules exhibited strong in vitro bioactivity, as evidenced by apatite formation in simulated body fluid (SBF), along with controlled degradation behaviour. Granules were made by converting clam shell calcium carbonate into calcium oxide through heat treatment, followed by wet chemical synthesis of calcium phosphate, polymer-assisted granulation, and high-temperature sintering. In vitro tests in simulated body fluid (SBF) showed rapid and uniform formation of an apatite layer on all samples, indicating strong osteoconductive properties. Phase and structural analysis showed that cooling conditions affected material properties, with air-cooled granules containing more α-tricalcium phosphate and degrading faster. Overall, the porous structure and ability to form apatite suggest good potential for bone growth and remodeling [71].

### 3.4. Scallop Shell-Based Biomaterials

Pramono et al. [72] investigated how ultrasonic processing time and calcination temperature affect the formation of nano-hydroxyapatite (nHAp) from scallop shells. XRD confirmed that hydroxyapatite formed successfully at 900 °C, with the best phase development at 60 min of sonication. FTIR results supported this by showing phosphate (PO_4_^3−^) and hydroxyl (OH^−^) peaks and the disappearance of carbonate signals, indicating full conversion of the scallop shell precursor. SEM and TEM showed evenly distributed nanoparticles, suggesting that ultrasonic treatment helped control grain growth. Biological tests showed that the scallop shell–derived nHAp was non-toxic and well tolerated by cells. Overall, these findings indicate that the integration of controlled sonochemical treatment with carefully optimized calcination conditions enables the efficient synthesis of phase-pure and biocompatible nHAp from scallop shell waste materials [72]. A subsequent study demonstrated that sonication plays a critical role in refining scallop shell-derived hydroxyapatite. X-ray diffraction (XRD) analysis showed that sonication produced a well-defined single-phase hydroxyapatite with a smaller crystallite size. In contrast, non-sonicated samples showed broader peaks and less uniform phase characteristics. SEM images revealed that sonicated powders had more uniform, ellipsoidal particles, while non-sonicated samples showed irregular shapes. XRF analysis also indicated higher compositional purity in the sonicated samples. FTIR confirmed hydroxyapatite functional groups in both cases, but non-sonicated samples showed additional peaks, suggesting incomplete phase formation. Overall, these results show that ultrasonic processing improves the structural quality and purity of nano-hydroxyapatite [93].

Syafaat and Yusuf [94] produced hydroxyapatite (HAp) from scallop shells using a precipitation method. The shells were first calcined at 1000 °C to form calcium oxide (CaO), then converted to calcium hydroxide and reacted with phosphoric acid, followed by a second calcination at 1050 °C to promote crystallization. XRD confirmed crystalline HAp formation, especially at higher Ca/P ratios. FTIR showed the expected phosphate and hydroxyl groups. SEM–EDX analysis indicated Ca/P ratios close to the ideal value (1.67) and showed that the calcium-to-phosphorus ratio affected particle shape and structure [94].

### 3.5. Cockle-Shell-Based Biomaterials

Zulkefli and coworkers [95] showed that cockle shell–derived HAp can be synthesized and used as a reinforcing phase in PLA-based composites for bone implant applications. Cockle shell waste was initially subjected to thermal treatment at 850 °C to decompose calcium carbonate (CaCO_3_) into calcium oxide (CaO). This was followed by a wet chemical precipitation process using phosphoric acid and ammonia. The product was then aged for 24 h, dried at 100 °C, and calcined at 900 °C to form crystalline hydroxyapatite. SEM–EDX and FTIR confirmed successful HAp formation with structural and compositional similarity to commercial standards. When incorporated into PLA at 10–30 wt.%, mechanical testing showed Young’s modulus values of 7.79–8.17 GPa, similar to cortical bone, with the highest stiffness at 30 wt.% HAp. These results show that cockle shell–derived calcium is a sustainable precursor for HAp/PLA biomaterials with good mechanical compatibility [95].

### 3.6. Mussel Shell-Based Biomaterials

Sathiyavimal et al. [96] designed a chitosan-reinforced hydroxyapatite scaffold derived from mussel shells and functionalized it with gentamicin, achieving a dual biological effect of enhanced osteoconductive activity and suppressed microbial growth. FTIR, XRD, and SEM analyses confirmed the successful formation of nanocrystalline hydroxyapatite embedded within a fibrous chitosan structure, creating a porous architecture that supports cell adhesion. In vitro tests with MG-63 osteoblast-like cells showed that the composite was non-toxic and significantly increased cell proliferation. Controlled release of gentamicin provided strong antibacterial action against certain osteomyelitis-associated pathogens, including *Staphylococcus aureus*, *Pseudomonas aeruginosa*, and *Klebsiella pneumoniae*. Integrated biopolymer scaffolds can provide antibiotic release to achieve simultaneous tissue regeneration and infection control [96].

El-Bassyouni et al. [85] investigated the production of nano-hydroxyapatite (nHAp) from mussel shells and characterized its physicochemical and biological properties for tissue engineering uses. Mussel shells were processed via wet chemical precipitation, with calcination at 900 °C to yield nanocrystalline HAp with a Ca/P ratio of ~1.68, which is similar to that of natural bone mineral. A comprehensive suite of techniques (XRD, SEM/EDX, TEM, FTIR, TGA, and XRF) confirmed high purity, nanoscale dimensions, and well-defined structural features. Biological tests, including cytotoxicity and proliferation assays using human mesenchymal stem cells, epithelial cells, and cancer cell lines, showed low toxicity and sustained cell growth over 7 days. Bovine serum albumin adsorption tests also showed strong protein binding, indicating good surface properties for protein interaction and cell attachment. Overall, these results suggest that mussel shell–derived nHAp is highly biocompatible and suitable for bone and soft tissue engineering applications [85].

Similarly, Ismail et al. [83] studied the reinforcement of biodegradable PLA/PCL scaffolds using mussel shell–derived hydroxyapatite to improve mechanical properties. Mussel shells were first calcined at 900 °C to convert calcium carbonate into calcium oxide, which was then reacted with a phosphate source using a wet precipitation method to form hydroxyapatite. XRD and FTIR confirmed the formation of pure-phase HAp. When added to PLA/PCL matrices, the composites showed evenly distributed particles and a porous structure. Increasing HAp content improved flexural strength, stiffness, and density, reaching values close to human cortical bone. In vitro degradation tests also showed controllable, composition-dependent breakdown behavior, supporting their use in orthopedic scaffolds [83].

Hydroxyapatite obtained from mussel shells has additionally been investigated as a bioactive surface coating for metallic implant materials. In the work by Kristianto et al. [97], calcium carbonate extracted from mussel shells was first converted into hydroxyapatite via a precipitation process and subsequently calcined at 1000 °C, producing a crystalline HAp phase with a Ca/P ratio of 1.63. This material was applied onto titanium alloy substrates using an electrophoretic dip-coating technique. Characterization using XRD, FTIR, SEM, and EDS confirmed the formation of single-phase hydroxyapatite and a consistent coating layer. Variations in processing parameters showed that higher voltages and increased calcination temperatures resulted in coatings that were more compact, thicker, and structurally uniform. The most effective conditions (50 V, 0.1 mm/s, 950 °C) yielded coatings with improved crystallinity and higher compressive strength, highlighting the usefulness of mussel shell–derived HAp for enhancing the surface properties of orthopedic titanium implants [97].

Collectively, these studies demonstrate that mussel shell–derived HAp is a biocompatible material suitable for the development of orthopedic scaffolds, polymer composites and implant coatings, and that these structural, mechanical, and biological properties can be further modified to achieve desired outcomes.

### 3.7. Oyster Shell-Based Biomaterials

Didekhani et al. [87] developed an electrospun composite scaffold composed of poly(L-lactide) (PLLA) and oyster shell powder to enhance stem cell osteoconductive responses for bone tissue engineering. Scaffold characterization by SEM and attenuated total reflectance Fourier-transform infrared spectroscopy (ATR-FTIR) revealed a fibrous area with interconnected pores and uniform shell particle distribution. The addition of oyster shell significantly increased tensile strength (from 1.17 to 1.72 MPa), indicating enhanced stability. Human mesenchymal stem cells isolated from adipose tissue (AT-MSCs) demonstrated favourable attachment and extensive spreading when seeded onto PLLA/OS scaffolds. Over a 14-day culture period, these scaffolds supported markedly improved cell growth and promoted greater levels of mineral deposition compared with the control conditions. Overall, the study demonstrated that electrospun PLLA/OS nanofibrous scaffolds improve mechanical properties, surface bioactivity, and cellular differentiation, supporting their potential use in bone tissue engineering applications [87].

In another multimaterial study, Luo et al. [98] fabricated 3D-printed scaffolds composed of polycaprolactone (PCL) and calcium carbonate from oyster shell powder using fused deposition modeling. Scaffolds containing 5 and 10 wt% oyster shell powder (OSP) exhibited highly regular, interconnected pore architectures and high porosity. SEM, FTIR, XRD, and DSC analyses confirmed that oyster shell powder (OSP) was evenly distributed throughout the PCL matrix without chemically altering the polymer. OSP also acted as a nucleation agent, increasing polymer crystallinity to about 58.6% and improving mineral formation. Mechanical testing showed that PCL/OSP scaffolds had compressive modulus values of 10–12 MPa, similar to cancellous bone. After 7 days in simulated body fluid (SBF), the composite formed more hydroxyapatite than pure PCL. In addition, MG-63 osteoblast-like cells showed increased proliferation and higher alkaline phosphatase (ALP) activity, indicating enhanced bone-forming response [98].

Calcium extracted from oyster shells has been further investigated as a viable raw material for designing composite coatings on metal implant surfaces. In this context, Hsieh et al. [86] engineered a composite coating consisting of nanohydroxyapatite (nHAp) integrated with peptides, applied onto pure titanium substrates, with the calcium precursor obtained from oyster shells. Oyster shell was calcined at high temperature (≈900–1000 °C) to produce calcium oxide, which was then hydrated to calcium hydroxide (Ca(OH)_2_) and used as the calcium source in a wet-chemical precipitation reaction with phosphate ions, yielding nanocrystalline hydroxyapatite. This oyster shell–derived nHAp was deposited as a coating on titanium substrates. XRD confirmed the formation of phase-pure hydroxyapatite, while FTIR confirmed the target ionic bands, verifying successful composite formation. SEM characterization revealed a surface morphology composed of interconnected nanofibrous and nanosheet-like features distributed evenly across the surface of the metal base. These findings support the conversion of oyster shell–derived material into a calcium precursor suitable as a biomimetic nHAp-based coating for implant surfaces [86].

Wu and coworkers [99,100] synthesized nHAp from oyster shells via a hydrothermal process by reacting oyster shell–derived calcium carbonate with dicalcium phosphate dihydrate through 5–10 h of ball milling and sintering at 1000 °C, where prolonged milling yielded phase-pure, highly crystalline HA. Phase and compositional analyses using XRD and FTIR verified a well-crystallized, single-phase nHAp structure with carbonate incorporation. Morphological examination by SEM indicated the presence of rod-shaped nanoparticles, with their aspect ratio positively correlated with increasing hydrothermal treatment time. In vitro SBF evaluation demonstrated that the hydrothermally processed sample formed the most homogeneous and continuous apatite layer resembling bone after 28 days of immersion. MC3T3-E1 pre-osteoblasts exhibited enhanced attachment and surface coverage on hydrothermally synthesized nHAp by day 7, confirming good cytocompatibility. The optimized formulation exhibited enhanced mechanical properties following sintering, including greater microhardness (~5.65 GPa) and improved fracture toughness (~1.23 MPa·m^0.5^), compared with the control sample [99,100].

In a similar study, Shen et al. [101] developed an injectable bone cement to address the impaired osteogenesis which is characteristic of diabetic nonunion. The cement consisted of a GelMA/HAMA hydrogel matrix integrated with glucose oxidase (GOx)–loaded mesoporous particles (nHAp) derived from oyster shell powder. The inclusion of nano-hydroxyapatite (nHAp) substantially strengthened the GelMA/HAMA composite network, while its gradual degradation enabled the gradual release of calcium and phosphate ions. In addition, nHAp functioned as a reservoir for glucose oxidase (GOx), supporting its controlled activation and subsequent local breakdown of excess glucose. Collectively, this bone cement system represents a promising strategy for addressing delayed fracture healing in diabetic environments by mitigating local hyperglycemia and associated inflammatory dysfunction [101].

The reviewed studies demonstrate that marine shell-derived biomaterials are highly versatile calcium-rich precursors capable of producing bioactive hydroxyapatite and composite biomaterials with properties closely resembling those of native bone mineral. Despite differences in shell origin, the majority of studies use similar processing strategies such as calcination followed by wet chemical precipitation or hydrothermal treatment. Across all marine shell-derived materials, enhanced osteoblast attachment, proliferation and osteogenic differentiation were repeatedly reported, highlighting the reproducibility of their osteoconductive potential [Table 1].

A notable trend throughout the literature is that the biological performance of marine shell-derived biomaterials depends more on material design than on the shell source itself. Incorporation into biodegradable polymers such as PLA, PCL, PLLA, and chitosan, as well as applications as implant surface coatings, consistently improved mechanical stability and cellular responses [61,87,95,96]. Likewise, optimization of particle size, crystallinity, porosity, and coating morphology enhanced apatite formation and promoted stronger interactions between the biomaterial and surrounding bone tissue [59,68,70,91]. These findings suggest that composite systems integrating shell-derived HAp provide greater performance than HAp alone. A similar pattern is observed in ES-based studies highlighted in the previous section. Among the marine-derived biomaterials, mussel- and oyster-derived hydroxyapatite demonstrated the broadest range of orthopedic applications, including porous scaffolds, polymer composites and implant coatings. In contrast, scallop- and cockle-derived materials have primarily been investigated as hydroxyapatite precursors and reinforcing phases, with comparatively fewer studies evaluating their biological performance in vivo. Overall, oyster- and mussel-derived biomaterials currently possess the strongest evidence supporting both mechanical enhancement and biological functionality. This trend likely reflects the greater maturity of research involving these oyster and mussel shell sources, their widespread availability as by-products of the seafood industry and the optimization of their processing methods, rather than evidence that they are intrinsically superior to scallop- or cockle-derived biomaterials.

Despite these promising findings, several limitations remain. Most studies focus on material synthesis, physicochemical characterization, and short-term in vitro evaluation, while none compare different marine shell sources under standardized experimental conditions. Considerable variation in processing methods, scaffold fabrication techniques, and biological outcome measures also limit direct comparison across studies. Future work should prioritize standardized preclinical evaluation, long-term degradation and remodeling studies, and direct comparisons between marine shell-derived biomaterials to identify the formulations with the greatest translational potential for clinical bone regeneration.

A comprehensive summary of studies developing eggshell- and marine shell-based biomaterials for orthopedic applications is presented in Table 1.

### 3.8. Current Status of ES and Bivalve-Derived Biomaterials

The above studies demonstrate that both avian- and aquatic-derived shell biomaterials represent sustainable calcium-rich sources capable of supporting bone regeneration. Regardless of their origin, most materials were successfully converted from calcium carbonate into hydroxyapatite or other bioactive calcium phosphate phases through calcination followed by wet precipitation or hydrothermal processing [Table 1]. These converted biomaterials consistently exhibited high phase purity, physicochemical properties comparable to native bone mineral, and favorable cytocompatibility, supporting osteoblast adhesion, proliferation, and differentiation.

Despite these common characteristics, distinct trends emerge between the two material groups. Eggshell-derived biomaterials have been investigated across the widest range of orthopedic applications, including metallic implant reinforcement, injectable hydrogels, porous scaffolds, implant coatings, and preclinical animal models [55,56,57,58,59,60,61,62,63]. Several studies further demonstrated success in vivo bone regeneration and, in some cases, clinical evaluation, highlighting the relatively advanced stage of ES-based biomaterial development [102]. In contrast, aquatic shell-derived biomaterials have predominantly focused on material synthesis, scaffold fabrication and implant surface coatings [101]. Although these materials consistently demonstrate excellent in vitro biocompatibility and mechanical performance, comparatively fewer studies have evaluated their regenerative capacity in vivo (Section 5). Another consistent observation is that biomaterial performance depends not only on the shell source but also on subsequent material engineering. Across both avian- and aquatic-derived biomaterials, the addition of biodegradable polymers including PLGA, PLA, PCL, chitosan, and silk fibroin, and optimization of HAp crystallinity, porosity, and particle size frequently resulted in superior biological and mechanical performance [58,61,71]. These modifications enhanced osteoconductivity, promoted apatite formation, improved degradation behavior, and increased mechanical compatibility with native bone, suggesting that composite and ion-substituted systems offer greater regenerative potential than unmodified hydroxyapatite alone. However, important limitations remain throughout the current literature. Considerable variation exists in synthesis protocols, calcination temperatures, particle size, scaffold architecture, and biological assessment methods, making direct comparison between studies difficult. Furthermore, most investigations remain limited to in vitro experiments or small-animal models (as presented in the following section), with relatively few studies evaluating long-term remodeling, load-bearing performance, or clinical translation. Standardized characterization methods and comparative in vivo studies will therefore be essential to determine which shell-derived biomaterials provide the optimal combination of bioactivity, mechanical performance, degradation behavior, and translational potential. Overall, the available evidence suggests that shell-derived biomaterials are promising alternatives to conventionally synthesized calcium phosphates. While eggshell-derived biomaterials currently possess the strongest preclinical and translational evidence, aquatic shell-derived materials demonstrate comparable physicochemical and biological characteristics and remain an underexplored resource with considerable potential for future orthopedic applications.

## 4. Animal Implant Models

**Figure 6:**
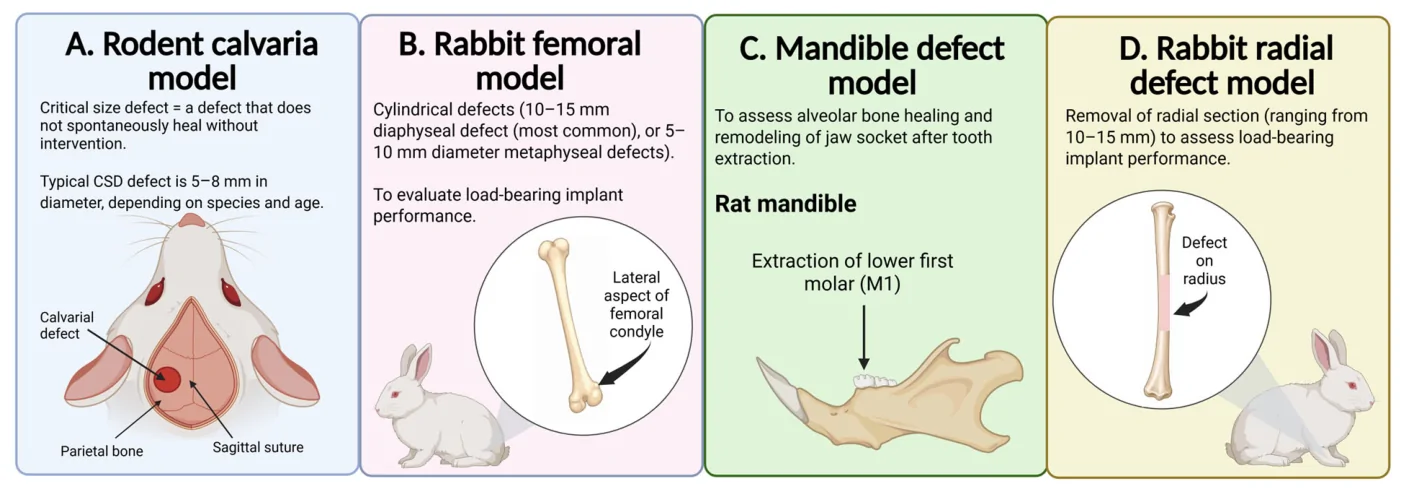
Animal models used to assess both load-bearing and non-load-bearing orthopedic implants. Original figure design and creation by S. Waqar.

Animal implant studies play a key role in bridging the gap between in vitro studies and clinical trials and provide valuable insight into the translational relevance of the implant (Figure 6) [103]. Bone studies using animal models include non-load-bearing (calvaria) and load-bearing (femur, mandible, tibia) implants, depending on the design and purpose of the implant (Figure 6).

One of the most widely used and well-established models for evaluating non-load-bearing bone implants is the critical-sized calvarial defect model [104]. Most commonly used in rats, the CSD can vary between 5 and 8 mm [104,105]. This model is typically implemented in rodents or rabbits and involves creating a controlled defect in the parietal bone of the skull to assess the regenerative potential of a biomaterial, scaffold, or therapeutic intervention [105]. The defect is designed to be of critical size, meaning it is large enough that it will not heal spontaneously during the lifetime of the animal, thereby requiring an intervention for regeneration to occur [105]. Therefore, new bone within the defect area can be directly linked to the implanted material or therapeutic intervention, which makes this model a robust and repeatable benchmark for assessing osteoconduction, osteoinduction, and overall bone regenerative capacity [103,106,107]. Its convenient anatomical accessibility provides consistent and well-controlled defect sizing, uniform surgical procedures, standardized imaging, and oriented histological evaluation. These features facilitate comparison between studies and underpin its widespread appeal for preclinical bone research [108].

In contrast, the rabbit radial and femoral model is widely used for studying load-bearing bone regeneration and fracture repair [109]. CSD in the rabbit femoral model can range from 10 to 15 mm diaphyseal defect (most common), or 5–10 mm diameter metaphyseal defects depending on application [109]. Similarly, CSD in rabbit radial defect ranges from 10 to 15 mm in size [109]. In this model, a controlled defect or fracture is created in the femoral diaphysis or metaphysis of a rabbit or the middle of the radius, and the site is treated with a biomaterial, scaffold, or therapeutic intervention [109]. Because the femur and radius are weight-bearing long bones, this model not only evaluates the material’s ability to promote bone formation but also allows assessment of mechanical stability, functional integration, and load-bearing performance [108,109]. The rabbit femoral model is commonly employed to examine implant fixation, interactions at the bone–implant interface, and overall mechanical strength, for translation to human long-bone fracture repair scenarios [110]. The suitable anatomical scale of this model enables precise surgical intervention, serial imaging, and biomechanical assessment, which are highly relevant to preclinical testing of orthopedic biomaterials [110].

In contrast, dental and craniofacial research generally relies on experimental models that replicate alveolar bone defects and tooth extraction scenarios, where localized regeneration and implant incorporation are the main outcomes [106]. The mandibular incisor extraction model is a preclinical platform to study alveolar bone repair, evaluate graft materials, and assess dental implant integration [102,109]. This model involves the surgical removal of one or more mandibular incisors, which generates a standardized and reproducible defect within the alveolar ridge that closely resembles clinical tooth loss [104]. The resulting site provides a controlled environment to evaluate the osteoconductive properties of biomaterials, scaffolds, growth factors, or other therapeutic interventions, as well as their biocompatibility and ability to support implant integration [108]. Because the surgical site is easily accessible, researchers can perform longitudinal monitoring using imaging techniques such as micro-computed tomography (micro-CT) or X-ray, as well as histological and histomorphometric analyses to quantify new bone formation [102,109]. The dental implant success model provides a framework for assessing functional bone healing, encompassing measures such as bone density, mineral content, and architectural stability [102,109]. It follows a reliable and clinically representative bone remodelling sequence, which makes it particularly useful for preclinical evaluation of new dental biomaterials intended to support and improve alveolar bone regeneration following tooth loss [109].

### 4.1. Preclinical In Vivo Assessment of Shell-Derived Bone Graft Substitutes

#### 4.1.1. Eggshell-Derived Bone Substitutes

Wu and coworkers [110] demonstrated that ES-derived porous biphasic calcium phosphate granules (EBGs) exhibit osteoconductive properties. The EBGs, composed of a 60/40 hydroxyapatite/β-tricalcium phosphate ratio, demonstrated high osteoblast viability in in vitro testing. In vivo implantation in a rabbit femoral defect model, evaluated by H&E-stained decalcified histology and micro-CT at 24 weeks, showed 23% filled bone volume compared with the commercial graft (Bicera—60% HA/40% β-TCP), which showed 18% filled bone volume, used as the control. These outcomes were attributed to the scaffold’s hierarchical porosity and the natural incorporation of osteogenic trace ions (Mg, Sr, Na, and Fe) derived from the eggshell precursor [110].

Acharjee et al. [111] carried out a comparative investigation of titanium-substituted hydroxyapatite scaffolds synthesized from eggshell waste and conventional laboratory-grade precursors for use in bone regeneration. Cleaned eggshell was thermally converted to calcium oxide, which was subsequently reacted with phosphoric acid, followed by the introduction of 5 wt% TiO_2_ as a dopant. In vitro assessments confirmed that all scaffold formulations were biocompatible, non-hemolytic, and supported high cell viability (>80%), while also demonstrating bioactive behaviour. In vivo evaluation using a rabbit femoral condyle defect model revealed improved osteogenesis in the eggshell-derived groups after two months, with Ti-doped ES-HAp demonstrating newly mineralized bone occupying 59% of the area within the defect. This value exceeded that of Ti-doped synthetic HAp (~54%), undoped ES-HAp (~43%), and undoped synthetic HAp (~31%) [111].

Salem et al. [112] found that eggshell-derived nano-hydroxyapatite (nHAp), especially when combined with zinc oxide nanoparticles, significantly improved bone repair in alveolar defects (Figure 6, Panel C). In a rat extraction socket model, nHAp treatment led to faster healing, increased bone formation, and better blood vessel formation compared with untreated sites and zinc oxide alone. Histology, SEM, and EDX analyses showed more organized, mineral-rich bone tissue, with the combined nHAp–ZnO treatment producing the best socket healing and bone quality [112].

Jayasree et al. [113] conducted in vitro and in vivo evaluations of an ES-derived brushite bone cement (EB) compared with a laboratory-synthesized brushite cement. ES-derived β-tricalcium phosphate was produced by calcining cleaned ES and reacting with monocalcium phosphate monohydrate to form brushite cement. The eggshell-derived (ES) cement contained trace ions such as Mg, Sr, Na, Si, and F, which together led to a longer setting time, a more gradual pH decrease, higher early compressive strength, and faster degradation compared with pure brushite. In vitro tests, including pH stability, degradation, compressive strength, MTT cell viability, and SEM cell attachment using L6 myoblasts and MG63 osteoblast-like cells, confirmed good biocompatibility and improved material performance. In a rat calvarial defect model (Figure 6—Panel A), the ES cement showed better healing, reduced inflammation, and complete defect closure by 12 weeks, outperforming pure brushite cement [113].

Wu et al. [114] investigated the osteoconductive performance of ES microparticles in protein-based hydrogels through implantation in critical-size defects (CSD) in Sprague-Dawley rats’ calvaria. When implanted into 10 mm CSD cranial defects in Sprague-Dawley rats, ES–reinforced GelMA scaffolds induced significant bone regeneration, achieving ~53–60 mm^3^ of new bone by 12 weeks—representing a ~6-fold increase over particle-less-GelMA and complete blank controls—along with bone densities (~2000 HU) comparable to native calvarial bone [114].

Later studies on composite systems investigated different apatite phases, trace-element doping, and eggshell-derived bone cements to improve bone regeneration. Hirota et al. [115] developed a magnesium-enriched hydroxyapatite paste from waste eggshells and tested its bone-forming ability using a periosteal pocket model in rat skulls. The eggshells were converted into a sulfate-free bio-apatite (BAp) through controlled ion exchange and calcination, producing a low-crystallinity, microcrystalline material enriched with magnesium and sodium. Material analysis showed that, compared to synthetic hydroxyapatite, the eggshell-derived apatite had smaller crystals, lower crystallinity, and released more ions. When implanted subperiosteally in PTFE tubes on rat calvaria, ES-derived apatite promoted significantly greater bone regeneration than conventional HAp, with ~55% higher bone mineral density as well as greater new bone area. Histological analysis further showed that ES-derived apatite was progressively resorbed and replaced by newly formed bone, whereas synthetic HAp largely persisted as residual material [115].

#### 4.1.2. Mussel Shell-Derived Bone Substitutes

Putra and coworkers [116] evaluated the osteoconductive potential of mussel shell–derived HAp in a rabbit femoral defect model. HAp was implanted into 5 mm metaphyseal defects and compared with commercially available bovine HAp and untreated controls. Bone healing was assessed using serum osteocalcin levels as a marker of osteoblast activity at 2, 4, and 6 weeks post-implantation. The mussel shell–derived hydroxyapatite group consistently showed higher osteocalcin levels at all time points compared with untreated defects. By week 2, levels were similar to those of the bovine HAp group and continued to increase through week 6. Histological analysis also showed a clear increase in osteoblast numbers by week 6 in the mussel shell HAp group, reaching levels comparable to bovine HAp and significantly higher than the control group [116].

In contrast, Trotta et al. [104] investigated whether mussel shell–derived powder could serve as a bone graft substitute in a 5 mm critical-size rat calvarial defect (Figure 6—Panel A). Cleaned mussel shell was ground to a particle size of 10–250 µm before sterilization and implantation. Histological evaluation at 30 and 90 days showed that, although the shell powder was biocompatible, it degraded quickly and did not effectively support bone formation. By 90 days, most of the defect area in the shell powder group was filled with fibrous connective tissue, with only about 7% of the defect area (mm^2^) filled with bone formation. In contrast, bovine bone particles remained in the defect and supported moderate bone formation (36% of defect area filled), while autografts achieved near-complete healing (89% of defect area filled). Adding mussel shell powder to bovine grafts did not improve outcomes, indicating little added benefit for bone repair. This was likely due to the small particle size and rapid degradation of the shell material, which limited long-term support for regeneration [104].

#### 4.1.3. Oyster Shell-Derived Bone Substitutes

Wang et al. [105] evaluated an oyster shell/α-calcium sulfate hemihydrate (α-CSH) scaffold combined with platelet-rich plasma (PRP) and bone mesenchymal stem cells (BMSCs) for bone regeneration. Before implantation, the scaffold underwent extensive characterization using SEM, FTIR, XRD, XRF, and simulated body fluid (SBF) testing, which confirmed a highly interconnected porous network. In vitro results showed that adding platelet-rich plasma (PRP) significantly increased BMSC proliferation, as indicated by higher alkaline phosphatase activity. In a 5 mm critical-sized calvarial defect model (Figure 6, Panel A) in Sprague–Dawley rats, micro-CT analysis after 8 weeks showed that the scaffold combined with both PRP and BMSCs produced the most new bone volume (1.71 ± 0.30 mm^3^). This was higher than the Scaffold + PRP group (1.25 ± 0.10 mm^3^), scaffold alone (0.87 ± 0.08 mm^3^), and untreated controls (0.83 ± 0.02 mm^3^). Histological analyses confirmed superior bone regeneration in the combined group, scaffold + PRP (38.72 ± 5.11%), scaffold-only (23.22 ± 2.35%), and control (14.19 ± 2.53%) groups, along with enhanced ALP and osteocalcin expression indicative of advanced bone maturation [105].

Ilham and coworkers [106] evaluated oyster shell as a precursor for bone graft material for post-extraction socket preservation. Oyster shell powder was milled into a fine calcium carbonate powder. Prior to implantation, characterization by FTIR confirmed a high calcium content. In vivo performance was evaluated using a guinea pig mandibular incisor extraction model, with comparisons to a xenograft positive control and an untreated negative control at 7, 14, and 21 days. Immunohistochemical analysis showed a decrease in RANKL expression in the oyster shell–treated group (from 8.00 ± 1.00 at day 7 to 2.67 ± 1.53 at day 21), comparable to the xenograft group and significantly lower than the negative control. Overall, the study concluded that oyster shell calcium carbonate effectively suppressed osteoclastogenic activity (prevents bone reabsorption), highlighting its potential as a low-cost, biocompatible material [106].

In another study, Yang and Zhang [103] investigated an oyster shell–derived nanocomposite material for bone defect repair using a bilateral rabbit radial bone defect model and compared it to an injectable calcium sulfate graft (Figure 6, Panel D). Oyster shell particles were processed to the nanoscale; a quantitative particle size distribution was not reported, and were mixed with an organic/polymeric matrix. Segmental radial defects of about 8–10 mm were created on both sides and evaluated at 2, 8, and 12 weeks after implantation. At 2 weeks, radiographs showed reduced bone mineral density and clear separation between graft and host bone in both groups, indicating limited early healing. By 12 weeks, the oyster shell–based material was fully integrated into the surrounding bone, while a gap remained between the graft and native bone in the control group. Mechanical testing showed higher radial bending strength in the oyster shell group (46.51 ± 1.58 MPa) compared with the calcium sulfate group (41.02 ± 1.26 MPa). In addition, quantitative colourimetric image analysis revealed a substantially greater extent of newly formed bone area in the experimental group (298.75 ± 35.12 µm^2^) compared with the control group (237.12 ± 21.03 µm^2^). Collectively, these results indicate that the oyster shell nanocomposite promoted superior graft integration, enhanced mechanical restoration, and greater osteoconduction than conventional calcium sulfate grafts in a rabbit segmental radial defect model [103].

#### 4.1.4. Cockle Shell-Derived Bone Substitutes

Punyanitya and coworkers [107] investigated hydroxyapatite (HAp)-based scaffolds derived from cockle shells. The shells were cleaned, calcined at 550 °C, milled, and reacted with ammonium dihydrogen phosphate, then sintered at 1100–1300 °C for 2 h. This produced a composite of HAp and β-tricalcium phosphate (β-TCP). XRD results showed that samples sintered at 1300 °C contained up to 91% hydroxyapatite, close to the purity needed for biomedical implants. SEM and porosity analysis showed a well-connected porous structure throughout the scaffolds. Mechanical evaluation indicated that increasing sintering temperature enhanced both hardness and flexural strength, producing values within the range of native bone tissue. Notably, in vivo subcutaneous implantation in rats demonstrated that scaffolds with greater hydroxyapatite content produced only minimal inflammatory reactions and maintained good tissue compatibility over a 90-day period, whereas scaffolds enriched in β-TCP underwent more extensive degradation. Collectively, these findings identified cockle shell as a suitable biocompatible calcium source for the fabrication of hydroxyapatite-based scaffolds [107].

### 4.2. Clinical Studies with Eggshell-Derived Biomaterial

Mupparapu et al. [102] conducted a randomized split-mouth clinical study to evaluate the effectiveness of eggshell-derived nano-hydroxyapatite (ES-nHAp) as a bone graft substitute for enhancing bone regeneration following surgical removal of mandibular third molars. Twelve patients were assessed clinically and radiographically over a six-month follow-up period, with wound healing, probing depth, bone density, trabecular pattern, and bone level compared between grafted and control sites. Sites treated with ES-derived nHAp exhibited smooth healing, along with higher bone density, enhanced trabecular architecture by month three, and decreased probing depths compared with control groups. No signs of infection were observed. These findings suggested that ES-derived nHAp was a safe, biocompatible, and effective synthetic graft material, which maintained bone height and promoted periodontal health, while offering a cost-effective and environmentally sustainable alternative to conventional graft substitutes [102].

A comprehensive summary of animal implant studies utilizing eggshell and marine shell material is presented in Table 2.

## 5. Conclusions and Future Perspectives of Naturally Derived Biomaterials for Bone Regeneration

**Figure 7:**
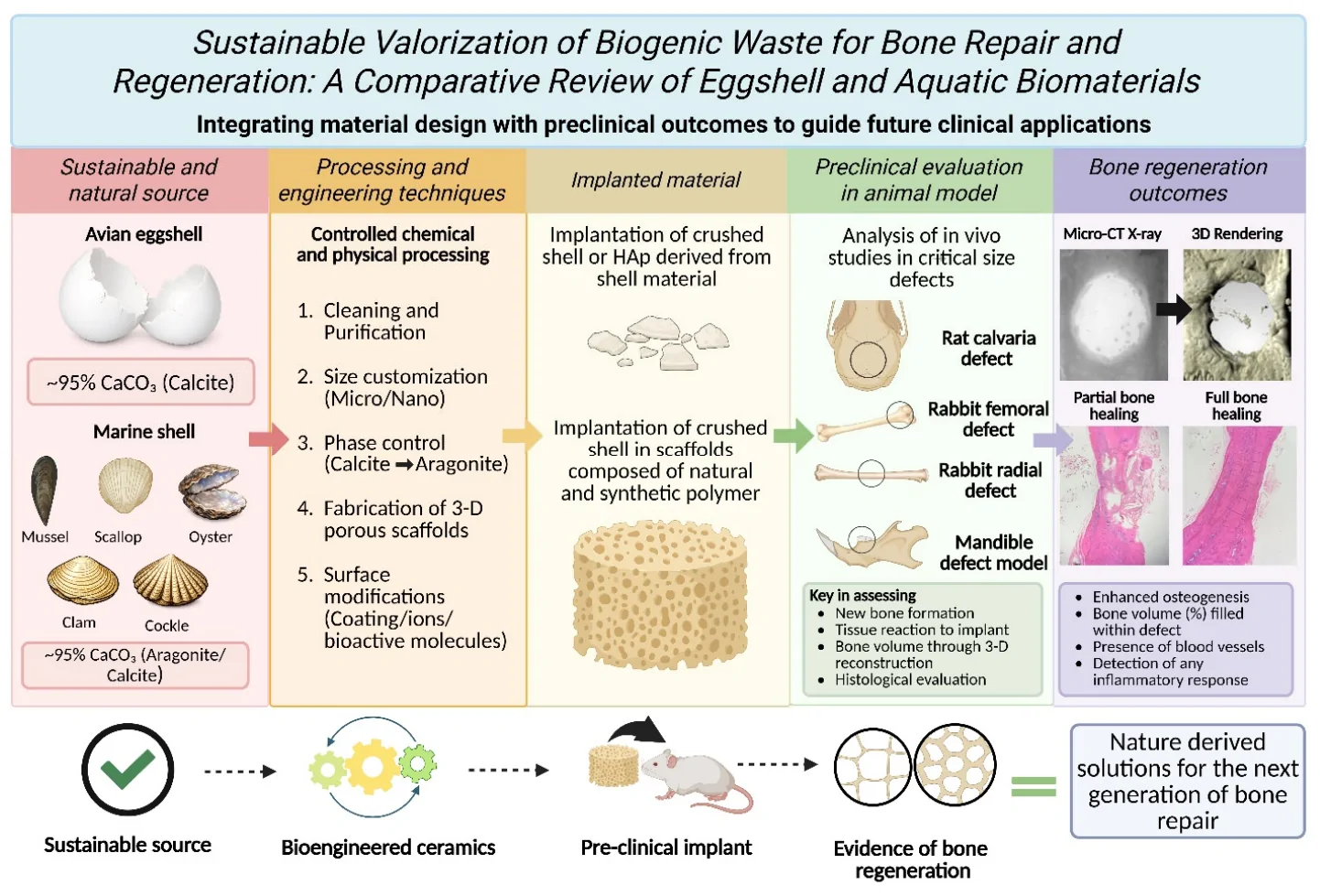
Overview of the sustainable valorization of eggshell and marine shell biowaste for bone repair and regeneration. The workflow illustrates the progression from natural calcium carbonate-rich shell sources through controlled chemical and physical processing, including purification, particle size customization, crystal phase modulation, scaffold fabrication, and surface functionalization. Processed shell-derived biomaterials are subsequently implanted as particulate grafts or polymer-based composite scaffolds and evaluated in preclinical animal models of critical-sized bone defects. Together, these approaches highlight the potential of shell-derived biomaterials as sustainable, bioengineered solutions for next-generation bone regeneration. Original figure design and creation by S. Waqar.

This review examined the structural and biological properties of biomaterials derived from avian eggshells and aquatic bivalve shells, focusing on research published between 2015 and 2025. Despite originating from different biological sources, these materials share a calcium-rich composition that makes them promising candidates for the development of osteoconductive bone substitutes (Figure 7).

Currently, conventional bone grafting materials from natural sources are most commonly bovine-derived xenografts [111,112,113]. Widely used materials such as Orthoss^®^, Bio-Oss^®^, Cerabone^®^, and Endobon^®^ consist primarily of a deproteinized inorganic matrix composed of HAp [108]. These materials are widely employed in dental and orthopedic applications for the repair of bone defects [108]. Their porous, trabecular morphology closely resembles native bone tissue, which is suitable for vascular ingrowth and the migration of cells to support new tissue development [112]. Due to their well-established performance, bovine-derived HAp materials are frequently employed as positive controls in studies evaluating alternative biomaterials, including eggshell- and bivalve-derived HAp [89,113].

ES-derived HAp has demonstrated promising biological performance in comparison to conventional graft materials. For example, a silicon (Si)-reinforced ES-derived HAp scaffold incorporated with poly(lactic-co-glycolic acid) (PLGA) exhibited a superior osteoblastic response (96%) relative to both bovine HAp/PLGA (90%) and synthetic HAp/PLGA (86%) controls [90]. In vivo studies using rabbit metaphyseal defect models showed that mussel shell–derived hydroxyapatite (HAp) increased osteocalcin levels at all time points compared with untreated defects. By week 2, these levels were similar to those seen with bovine-derived HAp and continued to rise by week 6. Histological analysis also showed a significant increase in osteoblast numbers in the mussel shell HAp group at week 6, comparable to bovine HAp and much higher than controls, indicating improved osteoblast recruitment and bone formation [110].

However, this trend is not consistently observed when minimally processed bivalve materials are used. In one study, cleaned mussel shells were ground to particle sizes of approximately 10–250 µm, sterilized, and directly implanted in critical size rat calvaria defect (5 mm). Although the material was biocompatible, it degraded quickly and showed limited ability to support new bone growth. Histological analysis at 30 and 90 days showed that most of the defect area was filled with fibrous tissue, with only about 7% new bone formation after 90 days. In contrast, bovine-derived bone particles remained at the defect site and supported greater bone regeneration, resulting in approximately 36% new bone formation. Combining mussel shell powder with the bovine graft did not improve outcomes beyond those achieved with the bovine graft alone. The lower performance of the mussel-derived material was mainly attributed to its small particle size and rapid degradation, which reduced its ability to provide long-term support for bone healing [104].

This review summarizes evidence of robust and reproducible bone regenerative outcomes using hydroxyapatite (HAp) derived from eggshell and bivalve shell sources. In rabbit femoral defect models, eggshell-derived HAp (ES-HAp) achieved ~23% bone volume regeneration, exceeding that of a commercial graft (Bicera—60% HA/40% β-TCP) (~18%) [99]. An additional improvement was reported for titanium-incorporated ES-HAp, which achieved approximately 59% new bone formation, in contrast to about 31% observed with non-doped synthetic HAp [112]. In rat critical-sized calvarial defect studies, ES-based brushite cement enhanced tissue repair while also diminishing inflammatory responses compared to empty controls [114]. In a mandibular defect model, eggshell-derived nano-hydroxyapatite (nHAp) promoted trabecular bone formation [113]. When combined with zinc oxide nanoparticles, it further improved healing in tooth extraction sockets compared with untreated sites [113]. Moreover, direct implantation of eggshell-derived calcium carbonate incorporated into a GelMA hydrogel led to increased bone mineral density in a rat calvarial defect model [114]. In a related approach, crushed oyster shell powder integrated into α-CSH scaffolds and supplemented with PRP and BMSCs significantly elevated new bone volume, with notable increases in alkaline phosphatase and osteocalcin expression [105].

Recent studies have reported many promising results for biomaterials derived from natural sources such as eggshells and bivalve shells [102,103,104,105,106,107,108,109,110,111,112,113]. These calcium-rich materials show potential for use in bone grafts, bone defect repair, and implant coatings [102,103,104,105,106,107,108,109,110,111,112,113]. However, differences in shell composition, processing methods, and biological performance highlight the need for greater standardization and more clinical studies. Further research is needed to support the development of sustainable, cost-effective, and biocompatible alternatives to conventional bone graft materials [108]. Due to their abundance and the large amounts of waste produced worldwide, eggshells and bivalve shells represent valuable resources for bone tissue engineering [108]. When processed into hydroxyapatite (HAp) and further optimized, these materials have strong potential as low-cost and sustainable alternatives for future orthopedic implants [109].

Hydroxyapatite exhibits strong osteoconductive properties, making it well-suited for bone and dental applications. Its biological and mechanical performance can be further improved through integration with other biomaterials such as β-tricalcium phosphate, platelet-rich plasma, PLGA, silk fibroin, alginate, or chitosan, all of which contribute to enhanced mechanical stability, bioactivity and regenerative capacity in defects [89]. While much of the current evidence is derived from in vitro studies and in vivo animal models, synthetic and shell-derived hydroxyapatite materials have also demonstrated promising clinical outcomes [106]. For instance, calcium phosphate systems combining hydroxyapatite and β-TCP have been successfully used in conjunction with bone marrow-derived mesenchymal stem cells in human clinical settings [105,111]. A European clinical study reported favorable outcomes using a 20:80 hydroxyapatite to β-TCP ratio for the treatment of long bone non-unions (including femur, tibia, and humerus), with radiological healing observed in a substantial proportion of cases (74% success rate and a 5% clinical criteria success rate) [107].

Despite these promising outcomes, further well-designed and long-term clinical trials are required to fully validate the effectiveness, reproducibility and translational applicability of naturally derived biomaterials for hard tissue regeneration. Eggshell- and bivalve shell- derived materials represent a sustainable and promising direction in bone tissue engineering, with strong potential for further development as next-generation orthopedic graft substitutes [114,115,116].

## Figures and Tables

**Table 1:** Comprehensive summary of recent in vitro studies (2015–2025) based on terrestrial (eggshell) and marine (clam, scallop, cockle, mussel, oyster) shell-derived biomaterials for orthopedic applications.

Shell Type (Derivative)	Application/Results	Processing Method	Limitations	Ref. No.
ES (calcium carbonate)	Crushed ES powder improved bovine osteoblast behavior in vitro.	CS	BV, NCE	[66]
	Orthopedic implants enhanced with ES-reinforced magnesium–yttrium composite alloys.	CS	BV, DBC	[67]
	Alginate–chitosan scaffold with crushed ES promoted osteogenic differentiation of stem cells in vitro.	CS	BV, MS	[60]
	Modified strontium-substituted wollastonite bioceramics show enhanced bioactivity and mechanical properties.	CS	DBC	[69]
ES (HAp)	HAp using calcination to create bone implant coating.	WCP	NCE	[59,68]
	HAp in combination with silk fibroin–alginate hydrogel for injectable dental application.	WCP	MS	[56]
	Europium-yttrium-doped HAp/chitosan composites enhanced bioactivities	WCP	MS	[58]
Clam Shell (HAp)	Hard clam and blood cockle clam shell-derived HAp as a potential biomaterial implant.	WCP	NCE	[88]
	HAp demonstrated strong bioactivity in SBF.	WCP	DBC	[89]
	HAp derived through a range of calcination temperatures.	WCP	NCE	[91]
	HAp combined with Ti-6Al-4V alloy substrates to develop an implant coating.	HP	NCE, DBE	[90]
	HAp combined with epoxy-based biocomposites to develop a ceramic reinforcement for load-bearing applications.	SSR	NCE, DBE	[92]
	nHAp combined with PLGA and chitosan.	WCP	MS	[61]
	nHAp demonstrated apatite formation in SBF and antibacterial activity.	WCP	MS	[84]
	Strontium-doped HAp demonstrated favorable osteoblast compatibility using MC3T3-E1 mouse cells.	HP	DBC	[70]
	Porous calcium phosphate granules exhibit strong in vitro bioactivity in SBF.	WCP	DBC	[71]
Scallop Shell (HAp)	nHAp via sonication and different calcination temperatures.	WCP	NCE	[72,93]
	HAp developed using a precipitation-based approach.	WCP	NCE	[94]
Cockle shell (HAp)	HAp combined with PLA-based composites.	WCP	MS, NCE	[95]
Mussel shell (HAp)	nHAp prepared using calcination.	WCP	NCE	[85]
	HAp combined with biodegradable PLA/PCL.	WCP	MS	[83]
	HAp used as a bioactive coating for titanium alloy substrates.	HP	NCE	[97]
	HAp scaffolds combined with chitosan and gentamicin to support bone growth.	WCP	MS	[96]
Oyster shell (nHAp)	nHAp combined with peptide coating for metal-based coating.	WCP	NCE, DBC	[86]
	nHAp derived from hydrothermal and ball milling process to enhance performance and mechanical strength.	HP	MS	[99,100]
Oyster shell (calcium carbonate)	3D-printed bone scaffolds composed of oyster shell powder and PCL using fused deposition model.	CS	BV, MS	[98]
	Oyster powder combined with PLLA through electrospinning to develop a composite for tissue replacement.	CS	BV, MS	[87]
	Injectable bone cement derived from oyster shell powder, GelMA and glucose oxidase particles.	CS	BV, DBC	[101]

Processing methods: WCP (Wet Chemical precipitation); HP (Hydrothermal precipitation); CS (Unmodified/Crushed shell), SSR (Solid state reaction); Limitations: MS (Mechanical strength); NCE (No cytocompatibility evaluation), DBC (Degradation behavior unclear), BV (Batch variability due to biological source).

**Table 2:** Comprehensive summary of clinical and in vivo studies using eggshell and bivalve shell-based material for the development of bone implant material. (Ref [102] is a human mandible study)

Species	Model	Description	Outcome	Evaluation Period	Ref. No.
Human	Mandibular	ES-nHAp as bone graft material.	Improved trabecular bone formation, without infection or adverse tissue reaction. Split-mouth control ***.	6 months	[102]
Rat		ES-nHAp combined with zinc oxide nanoparticles.	Accelerated socket healing, bone formation and enhanced vascularity. Control *.	4 weeks	[113]
Rabbit	Femoral model	Titanium-doped ES-derived HAp.	Superior bone regeneration (~59%). Control *.	2 months	[112]
		ES-derived porous hydroxyapatite/β-tricalcium granules.	Significantly (5%) more bone volume. Control *.	24 weeks	[111]
		Mussel shell-derived HAp.	Significant increase in osteoblast numbers and osteocalcin levels. Control **	2, 4, 6 weeks	[110]
	Radial defect model	Oyster shell nanoparticles combined with a polymer matrix.	Superior bone integration, greater new bone formation and higher mechanical strength. Control **	12 weeks	[103]
Rat	Critical size calvarial defect	ES-derived brushite bone cement.	Exhibited significantly higher healing scores, reduced inflammation, and complete bone bridging. Control *****.	12 weeks	[114]
		ES-reinforced GelMA scaffolds.	Induced significant bone regeneration, achieving ~53–60 mm^3^ of new bone as well as an increased bone density. Control ****	12 weeks	[115]
		Mussel shell–derived calcium carbonate particles (~10–250 µm).	Rapidly resorbed and failed to support osteoconduction due to the small particle size. Control *	30 and 90 days	[104]
		Oyster shell/α-CSH scaffolds combined with PRP and BMSC.	Enhanced bone volume along with enhanced ALP and osteocalcin expression. Control *	4 and 8 weeks	[105]
	Subcutaneous implantation	Cockle shell-derived HAp (high and low HAp content).	Elicited mild inflammatory responses and good tissue compatibility, and β-TCP–rich scaffolds exhibited greater resorption. No control.	7, 30 and 90 days	[107]

Note: The terms “bone volume” and “bone formation” are used interchangeably in some studies. In other studies, “bone area” and “bone regeneration” have similar meanings. Control: * (Empty defect/unfilled); ** (Bovine HAp: Bio-Oss or injectable calcium sulfate grafts); *** (Split mouth clinical control); **** Untouched bone; ***** Pure brushite cement.

## Data Availability

No new data were created or analyzed in this study. Data sharing is not applicable to this article.
